# A forward genetic screen identifies Sirtuin1 as a driver of neuroendocrine prostate cancer

**DOI:** 10.1084/jem.20241484

**Published:** 2026-05-28

**Authors:** Francisca Nunes de Almeida, Alessandro Vasciaveo, Arianna Giacobbe, Matteo Di Bernardo, Min Zou, Ainsley Mike Antao, Simone de Brot, Antonio Rodriguez-Calero, Alexander Chui, Alexander L.E. Wang, Nicolas Floc’h, Jaime Y. Kim, Soonbum Park, Stephanie N. Afari, Timur Mukhammadov, Nicholas Ortega, Sai Sampath Josyula, Juan Martin Arriaga, Jingqiang Wang, Jinqiu Lu, Michael M. Shen, Mark A. Rubin, Andrea Califano, Cory Abate-Shen

**Affiliations:** 1 Department of Molecular Pharmacology and Therapeutics, Vagelos College of Physicians and Surgeons, Columbia University Irving Medical Center, New York, NY, USA; 2 Department of Systems Biology, Vagelos College of Physicians and Surgeons, Columbia University Irving Medical Center, New York, NY, USA; 3 COMPATH, Institute of Animal Pathology, University of Bern, Bern, Switzerland; 4 Department of Biomedical Research, University of Bern, Bern, Switzerland; 5 Institute of Pathology, University of Bern and Inselspital, Bern, Switzerland; 6 Department of Biology, Columbia University, New York, NY, USA; 7 Department of Medicine, Vagelos College of Physicians and Surgeons, Columbia University Irving Medical Center, New York, NY, USA; 8 Department of Genetics and Development, Vagelos College of Physicians and Surgeons, Columbia University Irving Medical Center, New York, NY, USA; 9 Department of Urology, Vagelos College of Physicians and Surgeons, Columbia University Irving Medical Center, New York, NY, USA; 10 Herbert Irving Comprehensive Cancer Center, Columbia University Irving Medical Center, New York, NY, USA; 11 Department of Biomedical Research, University of Bern, Bern, Switzerland; 12 Bern Center for Precision Medicine, Bern, Switzerland; 13 J.P. Sulzberger Columbia Genome Center, Columbia University Irving Medical Center, New York, NY, USA; 14 Department of Biochemistry & Molecular Biophysics, Vagelos College of Physicians and Surgeons, Columbia University Irving Medical Center, New York, NY, USA; 15 Department of Biomedical Informatics, Vagelos College of Physicians and Surgeons, Columbia University Irving Medical Center, New York, NY, USA; 16 Chan Zuckerberg Biohub NY, New York, NY, USA; 17 Department of Pathology and Cell Biology, Vagelos College of Physicians and Surgeons, Columbia University Irving Medical Center, New York, NY, USA

## Abstract

Although most prostate tumors are relatively indolent, advanced disease can progress to aggressive, often lethal variants, including neuroendocrine prostate cancer (NEPC). To identify drivers of aggressive prostate cancer, we used *Sleeping Beauty* (*SB*) transposon mutagenesis in a mouse model having prostate-specific loss of *Pten* and *Tp53* (*NPp53* mice). Compared with control *NPp53-SB(−)* mice, experimental *NPp53-SB(+)* mice developed more aggressive tumors with increased metastasis. Notably, *NPp53-SB(+)* mice exhibited NEPC phenotypes with transcriptomic features that recapitulate human NEPC. Analysis of recurrent common insertion sites (CIS) and associated genes (CIS genes) identified genes differentially expressed between NEPC and non-NEPC tumors. Analysis of NEPC-enriched CIS genes by cross-species integration of genomic and transcriptomic data prioritized sirtuin 1 (*Sirt1*) as a candidate mechanistic determinant of NEPC. Gain- and loss-of-function studies in human prostate cancer cells and mouse NEPC organoids confirmed that *SIRT1* promotes NEPC, while pharmacological inhibition suppresses it. Thus, integration of cross-species analyses with an unbiased forward genetic screen uncovered novel drivers of NEPC.

## Teaser

Using an unbiased forward mutagenesis screen in an autochthonous mouse model, we have identified new mechanistic determinants of aggressive prostate cancer. *SIRT1* emerged as a key regulator of neuroendocrine prostate cancer differentiation and a potential target for therapeutic intervention.

## Introduction

Prostate cancer is the most common cancer in men, affecting one in six men in their lifetime. The vast majority present with regionally confined prostate adenocarcinoma that is often managed by active surveillance or local therapy, whereas for men with recurrent or advanced prostate cancer, the standard of care is androgen deprivation therapy (ADT) (Attard et al., 2015; Gelmann, 2002; Shen and Abate-Shen, 2010; Watson et al., 2015). Indeed, the androgen receptor (AR) is the most critical regulator of normal prostate differentiation as well as of all stages of prostate cancer progression (Abate-Shen and Shen, 2000; Gelmann, 2002; Shen and Abate-Shen, 2010). Consequently, prostate cancer treatments have been dominated by approaches to dampen AR signaling (Watson et al., 2015). However, while ADT initially leads to tumor regression, eventually tumors recur as castration-resistant prostate cancer (CRPC), so called because of its continued reliance on AR even in the absence of androgens (Scher and Sawyers, 2005). While further treatment of CRPC with second-generation anti-androgen therapies improves survival, many patients ultimately develop resistance and progress to aggressive disease variants that may no longer be dependent on AR (Watson et al., 2015). It is now well established that aggressive prostate cancer variants, including neuroendocrine prostate cancer (NEPC), arise through lineage plasticity (Beltran et al., 2016; Ku et al., 2017; Mu et al., 2017; Zou et al., 2017), defined as the transition from one differentiated cell state to another (Le Magnen et al., 2018). Thus, elucidating the mechanisms governing this process may improve treatment by overcoming plasticity-associated drug resistance.

We therefore sought to identify causal drivers of aggressive prostate cancer using a *Sleeping Beauty* (*SB*) forward genetics mutagenesis screen (Collier et al., 2005; Dupuy et al., 2005; Starr et al., 2009). *SB* is a two-component system composed of a mobile DNA transposon and a transposase enzyme that mediates transposon excision and random genomic insertion. Herein, biallelic mice carrying both the transposon and transposase are referred to as “active SB” mice (*SB^+^*), whereas mono-allelic mice carrying the transposase but lacking the transposon are designated “inactive SB” mice (*SB^–^*). When both the transposase and transposon are present—as in the *SB^+^* but not in the *SB^–^* mice—the transposon can insert randomly into the genome and induce mutations. Insertions in or near cancer-relevant genes that aberrantly activate or inactivate these genes may promote or modulate tumorigenesis.

Unlike CRISPR or RNAi-based screens, which target known genes and usually in a defined direction *(*i.e., either gain- or loss-of-function), *SB*-mediated transposition is an unbiased approach in which mutations can occur anywhere in the genome in both coding and noncoding regions, and may result in either gain- or loss-of-function of nearby genes (Beckmann and Largaespada, 2020; Copeland and Jenkins, 2010). Furthermore, because mutagenesis occurs in autochthonous mice, mutations arise somatically during the natural course of disease progression and within the native tumor environment, analogous to the accumulation of mutations as occur during human cancer progression. These features make *SB-*mediated mutational screens well suited for uncovering novel mechanisms of cancer progression (Dupuy et al., 2009), as demonstrated by studies in colorectal cancer (Iida et al., 2024; March et al., 2011; Shimomura et al., 2023; Starr et al., 2009; Starr et al., 2011) as well as tumors of the central nervous system (Beckmann et al., 2019), pancreas (Mann et al., 2012), melanoma (Mann et al., 2015), and prostate (Ahmad et al., 2016; Rahrmann et al., 2009). However, *SB* mutagenesis has been less widely adopted than RNAi or CRISPR approaches, in part because the random nature of transposon insertions complicates identification of the genes driving the resulting phenotypes.

In the current study, we performed a *SB* transposon–based screen in a well-characterized autochthonous genetically engineered mouse model (GEMM) of prostate cancer based on haploinsufficient loss of *Nkx3.1* and homozygous loss of *Pten* and *Trp53* (*NPp53* mice; Zou et al., 2017), modeling key genetic events frequently observed in advanced human prostate cancer (Abida et al., 2019; Cancer Genome Atlas Research, 2015). Compared with control *SB*-inactive *NPp53*-*SB(−)* mice, experimental *SB*-active *NPp53*-*SB(+)* mice develop more aggressive prostate cancer phenotypes with a high incidence of NEPC. Using an integrative systems biology approach—combining phenotypic, transcriptomic, and genomic analyses with state-of-the-art network-based algorithms—we have identified and validated mechanistic determinants of NEPC. Among these, we uncovered the nicotinamide adenine nucleotide (NAD)-dependent deacetylase *sirtuin 1* (*Sirt1*). We demonstrate that expression of *Sirt1* promotes NEPC while its silencing or pharmacological inhibition suppresses the NEPC phenotype. Overall, our study establishes a generalizable computational and experimental framework that integrates *SB* mutagenesis with phenotypic, genomic, and transcriptomic analyses to identify novel cancer drivers.

## Results

### Overall strategy

**Figure 1.**
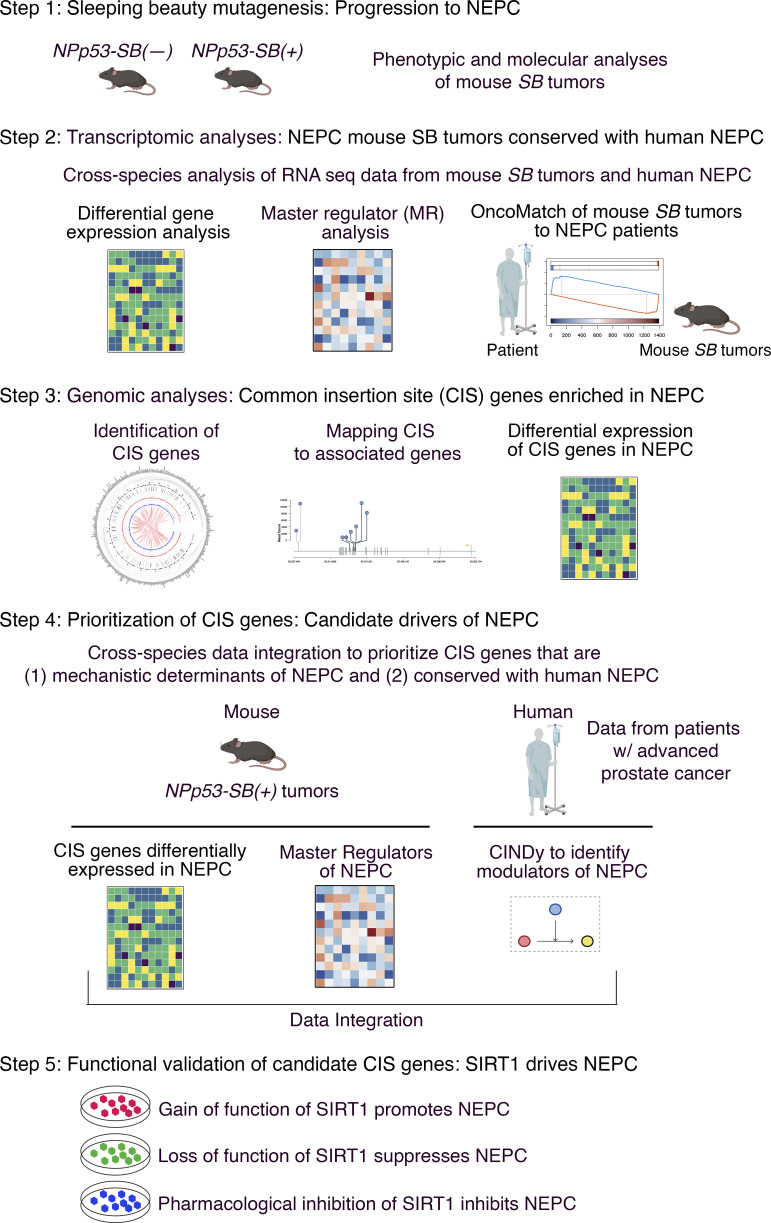
**Strategy to identify mechanistic determinants of NEPC by *SB* mutagenesis.** Shown is the overall strategy as described in the text. Step 1: *SB* mutagenesis was done on the *NPp53* mice GEEM of prostate cancer. *NPp53-SB(−)* and *NPp53-SB(+)* mice were generated, and phenotypic and molecular analyses were performed. Step 2: Transcriptomic data from *SB* mice were integrated with comparable data from human prostate cancer patient to assess conservation. Step 3: CIS of transposon integration were determined, and CIS genes were identified. Step 4: CIS genes were prioritized as candidate mechanistic determinants of NEPC by integrating transcriptomic and genomic data from mouse and human prostate cancer. Step 5: Candidates were functionally validated in human prostate cancer and mouse NEPC organoid models.

To identify mechanistic drivers of advanced prostate cancer, we used a *SB* mutagenesis screen, followed by integration of phenotypic, transcriptomic, and genomic analyses with systems biology algorithms. This integrative approach was combined with cross-species comparisons of mouse tumors and human cohorts to prioritize and functionally validate candidate genes (Fig. 1).

Step 1: We implemented the *SB* screen by crossing biallelic *SB* mice (Collier et al., 2005; Dupuy et al., 2005; Starr et al., 2009) with *NPp53* mice (Zou et al., 2017) to generate the experimental *NPp53-SB(+)* and the control *NPp53-SB(−)* mice (Fig. 1, step 1). Phenotypic analyses revealed that the *NPp53-SB(+)* mice had more aggressive prostate cancer phenotypes than the control *NPp53-SB(−)* mice with higher metastatic incidence and a greater prevalence of NEPC compared with controls.

Step 2: To evaluate their conservation with human NEPC, we performed transcriptomic analyses comparing histologically defined NEPC and non-NEPC tumors from *SB* mice (Fig. 1, step 2). Differential gene expression analyses of these RNA sequencing (RNA-seq) profiles confirmed that mouse tumors classified as NEPC were molecularly distinct from those classified as non-NEPC; further, these mouse NEPC tumors recapitulated established gene expression signatures of human NEPC. We applied the VIPER algorithm to convert these RNA-seq profiles into protein activity signatures (Alvarez et al., 2016) and to infer master regulators (MRs) that are candidate drivers of NEPC. Comparison of MR signatures from the mouse tumors with corresponding signatures of human NEPC using the OncoMatch algorithm (Alvarez et al., 2018; Mundi et al., 2023; Vasciaveo et al., 2023) revealed significant conservation. Together, these analyses demonstrate that the mouse *SB* NEPC tumors engage molecular programs that are highly conserved with human NEPC.

Step 3: To identify *SB* transposon integration sites, we performed genomic sequencing of the mouse *SB* tumors and applied Transposon Annotation Poisson Distribution Association Network Connectivity Environment (TAPDANCE) analysis (Sarver et al., 2012) to identify common insertion sites (CIS)—i.e*.,* those with statistically significant enrichment across multiple independent tumors (Fig. 1, step 3). We then mapped associated genes (“CIS genes”) and the location of the CIS within the genes. Differential gene expression analysis revealed a subset of CIS genes enriched in NEPC versus non-NEPC *SB* tumors.

Step 4: To identify CIS genes that represent candidate mechanistic determinants of NEPC, we implemented a cross-species integrative framework combining genomic and transcriptomic data from mouse and human NEPC tumors (Fig. 1, step 4). Specifically, integrating CIS mapping with MR transcriptomic analyses from mouse SB tumors, we applied the CINDy algorithm (Giorgi et al., 2014; Paull et al., 2021) to nominate CIS genes predicted to function as upstream modulators of NEPC MRs. Because such upstream modulators are more likely to exert causal control over transcriptional programs (Chen et al., 2014; Paull et al., 2021), this approach prioritized CIS genes with a high likelihood of driving the NEPC phenotype.

Step 5: Functional studies in human prostate cancer cell models and mouse organoid models using gain- and loss-of-function approaches in vitro and in vivo, as well as pharmacological inhibition, demonstrated that one of the top-ranked candidates, *Sirt1*, promotes NEPC (Fig. 1, step 5). Each of these steps is described in detail below.

### *SB* mutagenesis accelerates prostate cancer progression and promotes NEPC

**Figure 2.**
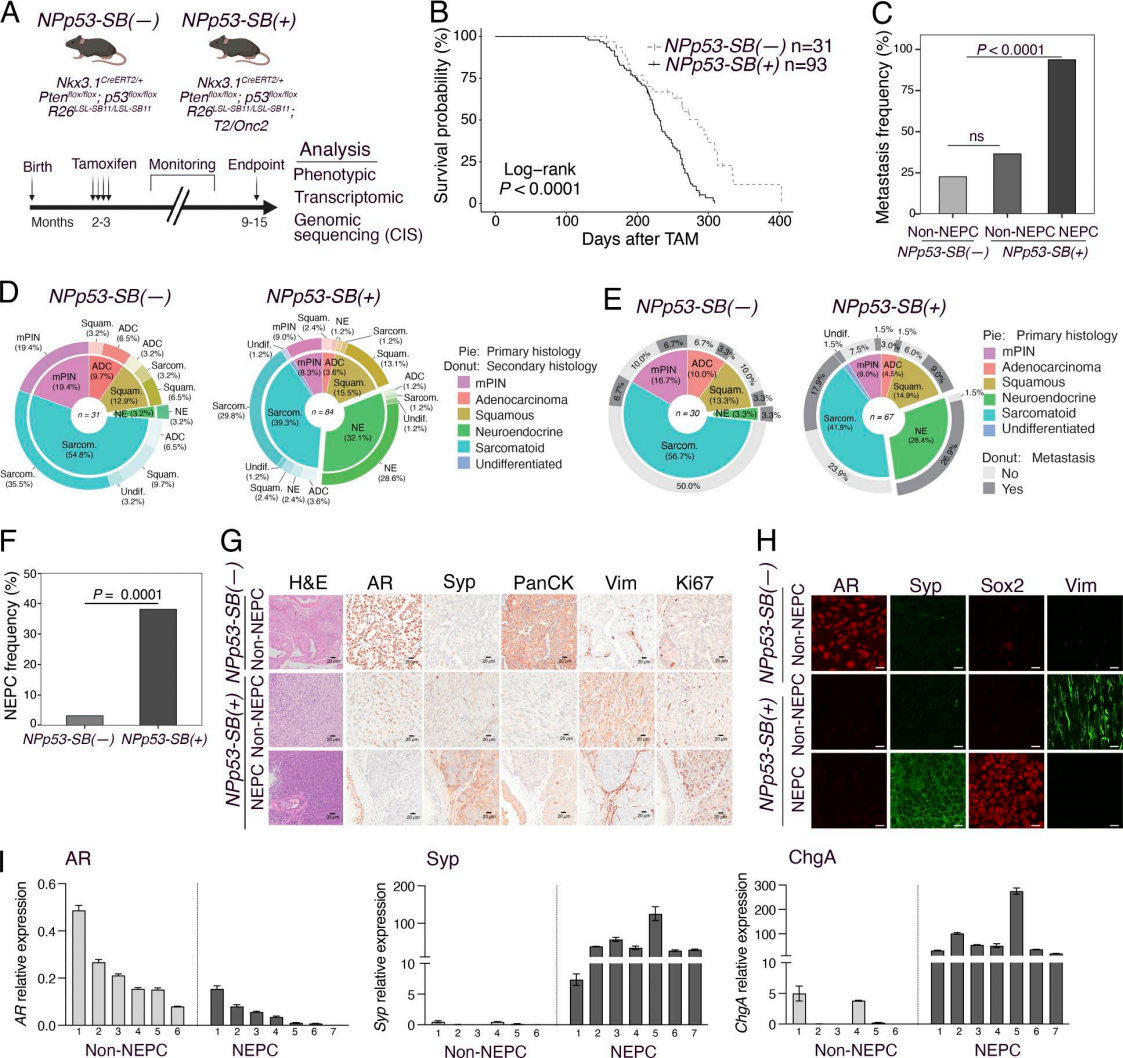
***SB* mutagenesis in *NPp53* mice promotes NEPC. (A)** Strategy: *NPp53-SB(−)* and *NPp53-SB(+)* mice (2–3 mo of age) were induced to form tumors by delivery of tamoxifen and the mice were monitored for up to 15 mo, following which tissues were collected for phenotypic (i.e., histological), transcriptomic (i.e., RNA-seq), and genomic (i.e., CIS) analyses. **(B)** Kaplan–Meier survival analysis of *NPp53-SB(−)* (*n* = 31) and *NPp53-SB(+)* (*n* = 93) mice. The P value was calculated using a Log-rank test. **(C)** Metastasis frequency in *NPp53-SB(−)* (*n* = 31) and *NPp53-SB(+)* (*n* = 84) mice comparing non-NEPC (*n* = 52) and NEPC (*n* = 32) tumors. The P value was calculated using the Fisher’s exact test. **(D and E)** Distribution of histological variant phenotypes and metastasis incidence in *NPp53-SB(−)* (*n* = 30) and *NPp53-SB(+)* (*n* = 67) mice. Histological phenotypes were classified as mouse prostatic intraepithelial neoplasia (mPIN), adenocarcinoma (ADC), squamous (Squam), neuroendocrine (NE), sarcomatoid (Sarcom), or undifferentiated (Undif). **(D)** Distribution of primary histology (pie chart) and secondary histology (donut chart). **(E)** Distribution of primary histology (pie chart) and metastatic status (donut chart). **(F)** NEPC frequency in *NPp53-SB(−)* (*n* = 31) and *NPp53-SB(+)* (*n* = 84) prostate tumors. The P value was calculated using the Fisher’s exact test. **(G and H)** Histopathological analyses. Representative images of prostate tumors from *NPp53-SB(−)* and *NPp53-SB(+)* mice, showing examples of NEPC and non-NEPC (adenocarcinoma and sarcomatoid) histopathology. Shown are H&E (G), and immunohistochemical (IHC; G) or immunofluorescence (IF; H) staining for markers of prostate cancer and NEPC, namely, AR, synaptophysin (Syp), Pan-cytokeratin (Pan-CK), vimentin (Vim), Ki67, and Sox2. Analyses were done on a minimum of five independent tumors for each group and each condition. Scale bars represent 20 µm for H&E and IHC, 10 µm for IF images. **(I)** Quantitative real-time PCR showing expression levels of *AR*, *Syp*, and *chromogranin A* (*ChgA*) in non-NEPC (*n* = 6) and NEPC (*n* = 7) *NPp53-SB(+)* tumors. Data are normalized using *Gapdh* as internal control. Analyses were done on six to seven independent samples with a minimum of two biological replicates. See also Fig. S1, Table 1, and Table S1.

We implemented the *SB* mutagenesis screen (Fig. 1, step 1) using the *NPp53* mice (*Nkx3.1*^*CreERT2/+*^*; Pten*^*flox/flox*^*; Trp53*^*flox/flox*^) (Fig. 2 A), a well-characterized autochthonous GEMM with prostate-specific loss of *Pten* and *Trp53* (Zou et al., 2017) that models prevalent genetic events in advanced human prostate cancer (Abida et al., 2019). Tumor induction is spatially and temporally controlled in the prostatic epithelium via the *Nkx3.1*^*CreERT2*^ allele (Wang et al., 2009b), which also confers haploinsufficiency for *Nkx3.1*, another common event in human prostate cancer (Cancer Genome Atlas Research, 2015). *NPp53* mice develop poorly differentiated prostate cancer that can progress to CRPC following androgen deprivation, while further treatment using second-generation anti-androgens promotes more aggressive phenotypes, including NEPC (Zou et al., 2017). Given this latent potential toward aggressive prostate cancer, we reasoned that *NPp53* mice would provide an ideal model to identify drivers of aggressive disease, including NEPC. As a control, we used the *NP* mice (*Nkx3.1*^*CreERT2/+*^*; Pten*^*flox/flox*^; Floc’h et al., 2012), which develop well-differentiated prostate adenocarcinoma that progresses to CRPC but does not advance to more aggressive phenotypes following androgen deprivation or anti-androgen treatment (Zou et al., 2017).

**Figure S1.**
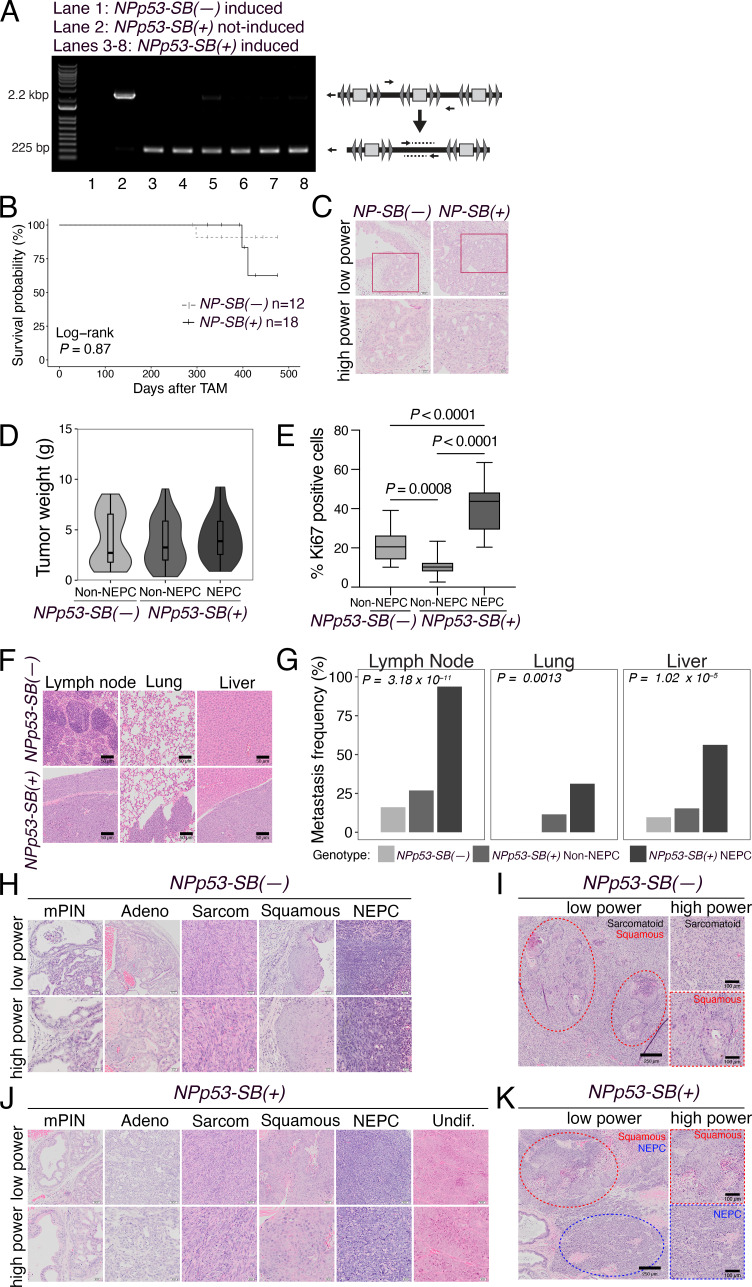
**Additional analyses of the prostate phenotype of *SB* mice (related to Fig. 2**). **(A)** PCR showing evidence of excision of the *T2/Onc2* transposon in induced *NPp53-SB(+)* tumors (lanes 3–8) but not in tumors without the *SB* (*NPp53-SB(−)*) or non-induced *NPp53-SB(+)* tumors (lanes 1, 2, respectively) (*n* = 10). **(B and C)** Analysis of the phenotype of *NP-SB(−)* (*n* = 12) and *NP-SB(+)* mice (*n* = 18). **(B)** Kaplan–Meier survival analysis. P value was calculated using a Log-rank test. **(C)** Representative H&E images of prostate tumors showing low power and high-power. *n* = 5 independent tumors/group. Scale bars represent 50 µm for low power and 20 µm for high power H&E. **(D and E)** Additional analyses of non-NEPC and NEPC tumors from the *NPp53-SB(−)* and *NPp53-SB(+)* mice. **(D)** Summary of tumor weights from *NPp53-SB(−)* (*n* = 29) and *NPp53-SB(+)* non-NEPC (*n* = 43) and *NPp53-SB(+)* NEPC (*n* = 27) mice. **(E)** Quantification of Ki67 expression in tumors from *NPp53-SB(−)* and *NPp53-SB(+)* non-NEPC and NEPC. Data were obtained by counting a minimum of 8,000 cells from five sections per tumor from three independent tumors per condition. **(F and G)** Analyses of metastasis in *NPp53-SB(−)* and *NPp53-SB(+)* mice. Representative cases of *NPp53-SB(−)* (*n* = 29) and *NPp53-SB(+)* non-NEPC (*n* = 43) and *NPp53-SB(+)* NEPC (*n* = 27) mice. **(F)** Representative H&E images of the indicated tissues. Scale bars represent 50 µm. **(G)** Metastasis frequency for the tissues indicated. **(H–K)** Representative H&E images showing examples of variant tumor histology for *NPp53-SB(−)* (*n* = 29) and *NPp53-SB(+)* non-NEPC (*n* = 43) and *NPp53-SB(+)* NEPC (*n* = 27) mice. **(H and I)** Selected *NPp53-SB(−)* cases. **(J and K)** Selected *NPp53-SB(+)* cases. Scale bars in H and J represent 50 µm for low power (top) and 20 µm for high power (bottom) images. Scale bars in I and K represent 250 µm for low power (left side) and 100 µm for high power (right side). Detailed histopathological analysis is provided in Table S1. Source data are available for this figure: SourceData FS1.

The *SB* system is comprised of two alleles: a conditionally activatable transposase in the *Rosa26* locus (*Rosa26*^*LSL-SB11*^) and a transgenic transposon allele (*T2/Onc2*) (Collier et al., 2005; Dupuy et al., 2005; Starr et al., 2009). Cre activation via the tamoxifen-inducible *Nkx3.1*^*CreERT2/+*^ excises the stop cassette blocking *SB11* expression, enabling transposase expression specifically in prostatic epithelium. The transposase then recognizes the terminal repeats of T2/Onc2, promoting its excision and random genomic integration (Dupuy et al., 2005; Starr et al., 2009). Therefore to generate the relevant *SB* mice, we crossed the *Rosa26*^*LSL-SB11*^ and*T2/Onc2* alleles with *NPp53* mice to produce experimental *NPp53-SB(+)* mice (*Nkx3.1*^*CreERT2/+*^*; Pten*^*flox/flox*^*; Trp53*^*flox/flox*^*; Rosa26*^*LSL-SB11/LSL-SB11*^*; T2/Onc2*), which carry both transposase and the transposon, and control *NPp53-SB(−)* mice (*Nkx3.1*^*CreERT2/+*^*; Pten*^*flox/flox*^*; Trp53*^*flox/flox*^*; Rosa26*^*LSL-SB11/LSL-SB11*^), which lack the transposon (Fig. 2 A). Tamoxifen induction of *NPp53-SB(+)* mice results in Cre-mediated activation of the transposase, resulting in *T2/Onc2* transposition, as confirmed by PCR (Fig. S1 A), whereas *NPp53-SB(−)* mice have no transposition (Fig. S1 A). For comparative analyses, corresponding *NP-SB(−)* mice (for *Nkx3.1*^*CreERT2/+*^*; Pten*^*flox/flox*^*; Rosa26*^*LSL-SB11/LSL-SB11*^) and the *NP-SB(+)* mice (for *Nkx3.1*^*CreERT2/+*^*; Pten*^*flox/flox*^*; Rosa26*^*LSL-SB11/LSL-SB11*^*; T2/Onc2*) were also generated and analyzed (Fig. S1, B and C).

We performed tamoxifen-mediated tumor induction of *SB* mice at 2–3 mo of age and monitored them for up to 12 mo or until they succumbed to prostate cancer, after which prostate tumors and other tissues were collected for phenotypic and molecular analyses (Fig. 2 A). Compared with the *NPp53-SB(−)* mice, the *NPp53-SB(+)* mice developed more aggressive prostate cancer, as evidenced by their histology, reduced survival, and increased metastasis (Fig. 2, B–I; Fig S1, D–K;Table 1, and Table S1). Specifically, the *NPp53-SB(+)* mice displayed a significant reduction in survival relative to the *NPp53-SB(−)* mice (log-rank P value <0.0001; Fig. 2 B). In contrast, the *NP-SB(+)* mice showed no significant decrease in survival or acceleration of their prostate cancer phenotype compared with the *NP-SB(−)* mice (log-rank P value = 0.87; Fig. S1 B, Table 1, and Table S1), consistent with prior observations that they do not progress to advanced disease (Zou et al., 2017). *NPp53-SB(+)* mice also showed markedly higher metastatic incidence to lymph nodes, lung, and liver, predominantly in mice with NEPC tumors (P < 0.0001; Fig. 2, C–E; Fig. S1, F and G; and Table 1). These findings indicate that *SB* mutagenesis of *NPp53* tumors promotes highly aggressive, metastatic prostate cancer with frequent NEPC, arising de novo in hormonally intact, untreated mice.

**Table 1.** Summary of the mouse models used in this study and their associated phenotypes

Name	Genotype	N	Histological phenotype	Mets	NEPC
*NPp53-SB(−)*	*Nkx3.1* ^*CreERT2/+*^ *; Pten* ^*flox/flox*^ *; Trp53* ^*flox/flox*^ *; R26* ^*LSL-SB11/LSL-SB11*^	31	Poorly differentiated adenocarcinoma with sarcomatoid features	7/31 (22%)	1/31 (3%)
*NPp53-SB(+)*	*Nkx3.1* ^*CreERT2/+*^ *; Pten* ^*flox/flox*^ *; Trp53* ^*flox/flox*^ *; R26* ^*LSL-SB11/LSL-SB11*^ *; T2/Onc2*	93	Poorly differentiated adenocarcinoma with sarcomatoid features and neuroendocrine differentiation	57/93 (61%)	32/84 (38%)
*NP-SB(−)*	*Nkx3.1* ^*CreERT2/+*^ *; Pten* ^*flox/flox*^ *; R26* ^*LSL-SB11/LSL-SB11*^	12	High-grade PIN, well-differentiated adenocarcinoma	0/12 (0%)	0/12 (0%)
*NP-SB(+)*	*Nkx3.1* ^*CreERT2/+*^ *; Pten* ^*flox/flox*^ *; R26* ^*LSL-SB11/LSL-SB11*^ *; T2/Onc2*	18	High-grade PIN, well-differentiated adenocarcinoma	0/18 (0%)	0/18 (0%)

Mets, metastasis; NEPC, neuroendocrine prostate cancer; PIN, prostatic intraepithelial neoplasia.

As is characteristic of *NPp53* mice (Zou et al., 2017), histopathological analyses of *NPp53-SB(−)* and *NPp53-SB(+)* prostate tumors revealed heterogeneous phenotypes (Fig. 2, D, E, G, and H; Fig. S1, H–K;Table 1, and Table S1). However, while *NPp53-SB(−)* tumors were predominantly squamous or sarcomatoid, similar to the baseline *NPp53* tumors (Zou et al., 2017), a significant fraction of *NPp53-SB(+)* tumors displayed NEPC features, which are more rarely seen in baseline *NPp53* tumors (P < 0.0001; Fig. 2 F, Table 1, and Table S1). Based on previously published histopathological criteria (Ittmann et al., 2013; Shappell et al., 2004), NEPC in mouse prostate tumors was classified based on characteristic histopathological features, including (1) nesting or trabecular growth patterns, (2) small cells with scant cytoplasm, and (3) high mitotic activity (Table S1). Further, tumors classified as NEPC in *NPp53-SB(+)* mice expressed canonical NEPC markers—Sox2, synaptophysin (Syp), chromogranin A (ChgA), and Sox2—alongside reduced AR expression (Fig. 2, G–I). Despite comparable tumor weights (Fig. S1 D), NEPC tumors were significantly more proliferative than non-NEPC tumors, as shown by Ki67 staining (P < 0.0001; Fig. 2 G and Fig. S1 E).

### NEPC tumors from *NPp53-SB(+)* mice recapitulate molecular programs of human NEPC

**Figure 3.**
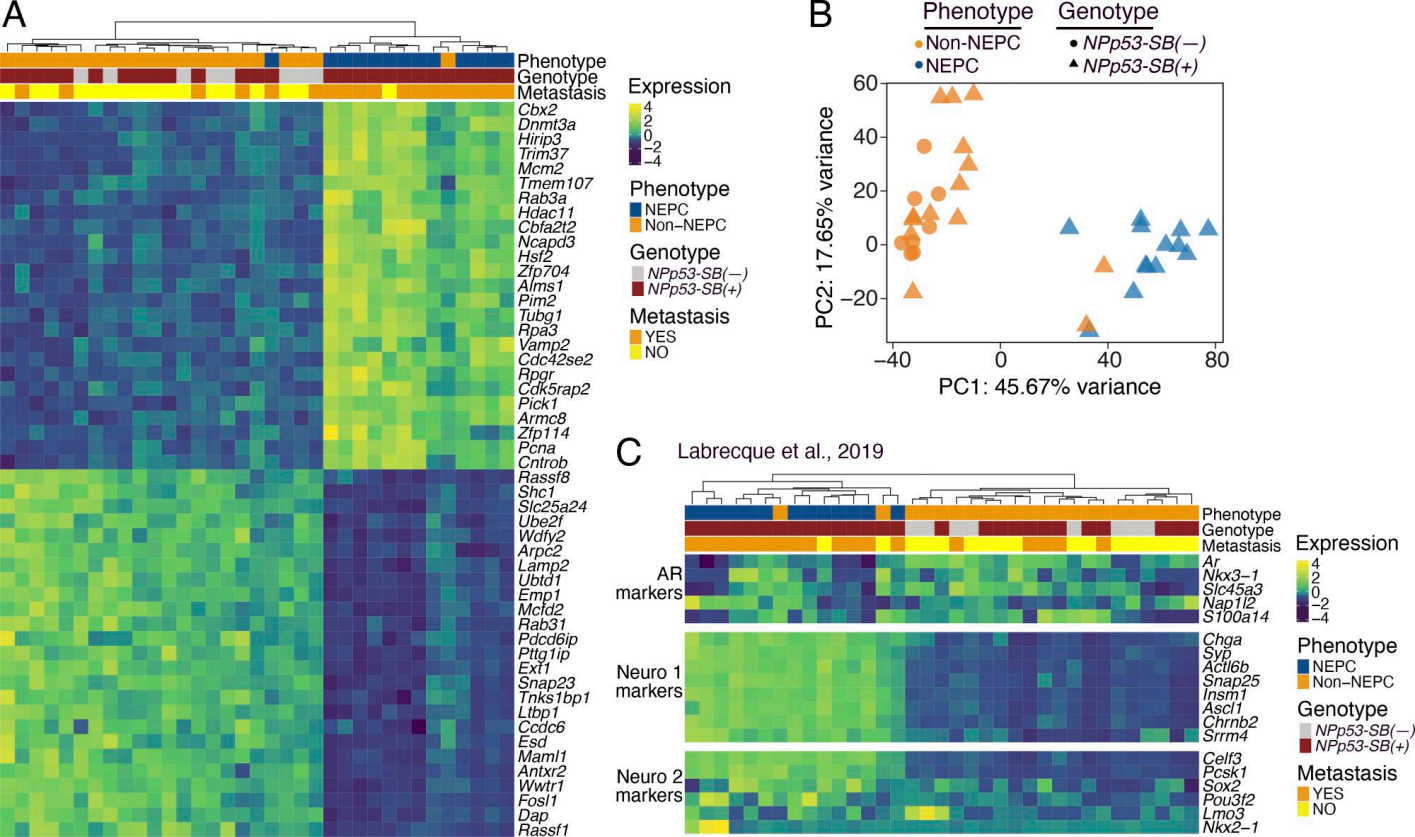
**Transcriptomic analysis of *SB* mouse prostate tumors.** Analyses of RNA-seq data from histologically defined non-NEPC and NEPC tumors from *NPp53-SB(−)* (*n* = 8) and *NPp53-SB(+)* (*n* = 27) mice. **(A)** Unsupervised clustering visualized by heatmap analyses. Shown are the top 25 and bottom 25 differentially expressed genes. **(B)** PCAs. **(C)** Heatmap showing relative expression of mouse orthologs of gene markers associated with AR-driven and NEPC phenotypes, as defined in human tumors (Labrecque et al., 2019). Phenotype, genotype, and metastatic status are indicated above. See also Tables S1 and S2.

To characterize their transcriptomic phenotype (Fig. 1, step 2), we performed RNA-seq on NEPC or non-NEPC tumors from *NPp53-SB(+)* or *NPp53-SB(−)* mice (*n* = 27 and *n* = 8, respectively; Fig. 3, A–C; and Table S2 A). Differential gene expression and principal component analyses (PCAs) revealed that NEPC *NPp53-SB(+)* tumors clustered separately from both non-NEPC *NPp53-SB(+)* tumors and *NPp53-SB(−)* tumors (Fig. 3, A and B), indicating their distinct transcriptional state. Notably, genes that are differentially expressed in NEPC *SB* tumors included those linked to cancer progression or neuroendocrine differentiation in humans, such as *RASSF1*, which is down-regulated in the NEPC *SB* tumors and is frequently silenced by promoter hypermethylation across multiple malignancies, including prostate cancer (Liu et al., 2002), and *CBX2*, which is up-regulated in the NEPC *SB* tumors and in human prostate cancer has been shown to be associated with metastasis and poor clinical outcome (Clermont et al., 2016).

To directly assess their similarity with human NEPC, we compared RNA-seq profiles from mouse *SB* tumors with well-characterized gene signatures of human AR-driven and neuroendocrine CRPC (Labrecque et al., 2019). Unsupervised clustering revealed that mouse NEPC *SB* tumors were enriched for signatures of known NEPC subtypes, namely NEURO I and NEURO II, and have correspondingly reduced AR-related gene expression, whereas non-NEPC *SB* tumors displayed the opposite pattern (P < 0.05; Fig. 3 C and Table S2 B).

**Figure 4.**
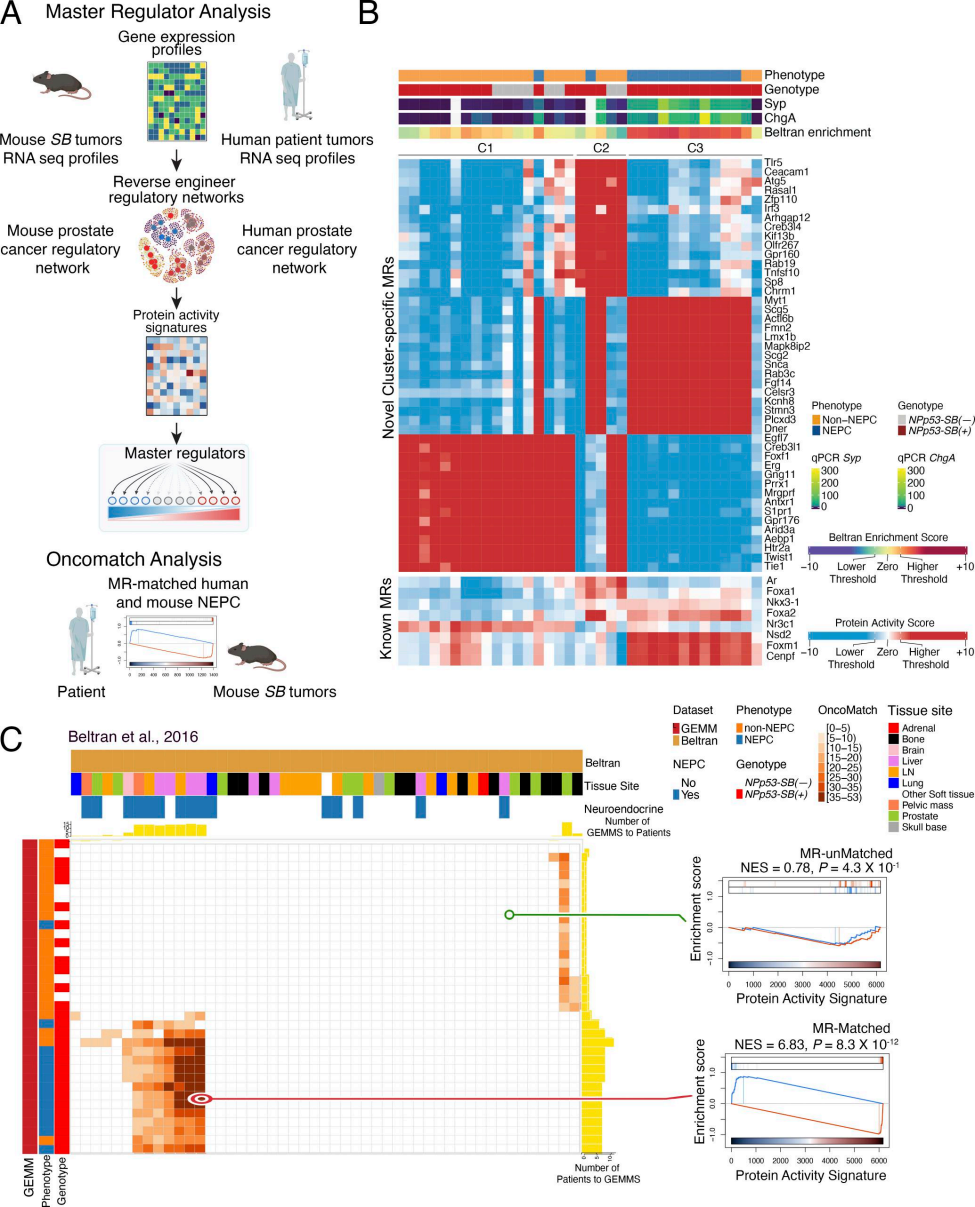
**MR profiles of mouse NEPC are conserved with human NEPC. (A)** Strategy. MR analyses: Differential gene expression profiles were converted to protein activity profiles which were then used to identify MRs of NEPC. Analyses were done using species-specific (i.e., mouse or human) regulatory networks as in Vasciaveo et al. (2023). OncoMatch: Individual mouse MR signatures were compared with individual human MR signatures. **(B)** Heatmap of protein activity-based cluster analysis of non-NEPC and NEPC mouse *SB* prostate tumors. Projected are the top 15 MRs that distinguish each cluster. Also shown are the activity levels of known MRs of advanced prostate cancer and NEPC. Shown at the top are the phenotype and genotype, quantitative real-time gene expression levels for *synaptophysin* (*Syp*) and *chromogranin A* (*ChgA*), and enrichment of the Beltran NEPC signature reported in Beltran et al. (2016). **(C)** OncoMatch heatmap representing conservation of MRs between individual *SB* tumors (rows, *n* = 35 samples from 32 independent tumors) and individual patient tumors (columns, *n* = 49) from the Beltran cohort. Patient features (i.e., assessment of neuroendocrine features and tissue site) are shown on the top horizontal bars, while GEMM features (i.e., phenotype and genotype) are shown on the left side vertical bars. Yellow bars at the top of the heatmaps show the number of GEMMs that match each patient, while the yellow bars on the right side show the number of patients that match each GEMM. The gene set enrichment analysis on the left show representative *SB* tumor and human patient pairs showing an example of an MR-unmatched (low-fidelity, top) and an MR-matched (high-fidelity, bottom) pair. See also Fig. S2, Table S3, and Table S4.

To extend these findings to protein activity-based analysis, we applied the VIPER algorithm (Alvarez et al., 2016) to infer differential protein activity from gene expression signatures of the NEPC and non-NEPC *NPp53-SB(−)* and *NPp53-SB(+)* tumors (Fig. 4 A and Table S3 A), using a mouse prostate cancer interactome previously constructed from murine tumor RNA-seq data (Vasciaveo et al., 2023). Protein activity– based clustering identified three molecularly distinct subtypes (*C*_*1*_–*C*_*3*_) composed predominantly of NEPC or non-NEPC tumors (Fig. 4 B and Table S3 B). Subtypes *C*_1_ and *C*_2_ consisted primarily of *NPp53-SB(−)* tumors and non-NEPC *NPp53-SB(+)* tumors, whereas subtype *C*_*3*_ was composed primarily of NEPC *NPp53-SB(+)* tumors. Notably, *C*_*3*_ tumors exhibited robust expression of canonical NEPC markers, including *Syp* and *ChgA*, as confirmed by quantitative PCR (Fig. 2 I and Fig. 4 B). Further, proteins differentially active in human NEPC—derived from a signature of metastatic CRPC patients that have both CRPC-adenocarcinoma (CRPC-adeno) and NEPC (CRPC-NE) (Beltran et al., 2016)—were significantly enriched among proteins differentially active in *C*_*3*_ tumors relative to *C*_*1*_ and *C*_*2*_ (Fig. 4 B).

The proteins inferred to be most differentially active in *C*_*3*_ tumors, which represent candidate MRs of NEPC, include those previously implicated in NEPC, such as Myt1 (Fig. 4 B) (Guo et al., 2019). Notably, 13 of the top 15 MRs in *C*_*3*_, including Lmx1b, Dner, Snca, and Rab3c, have functions associated with neural development or nervous system function (Ding et al., 2003; Fischer von Mollard et al., 1994; Han et al., 2021; Hsieh et al., 2013; Siddiqui et al., 2016). In addition, the *C*_*3*_ tumors exhibited high activity of MRs linked to aggressive prostate cancer, including Foxm1 and Cenpf, as well as NEPC regulators, including Nsd2, Foxa2, and Foxa1 (Fig. 4 B) (Han et al., 2022; Li et al., 2026; Vasciaveo et al., 2023).

**Figure S2.**
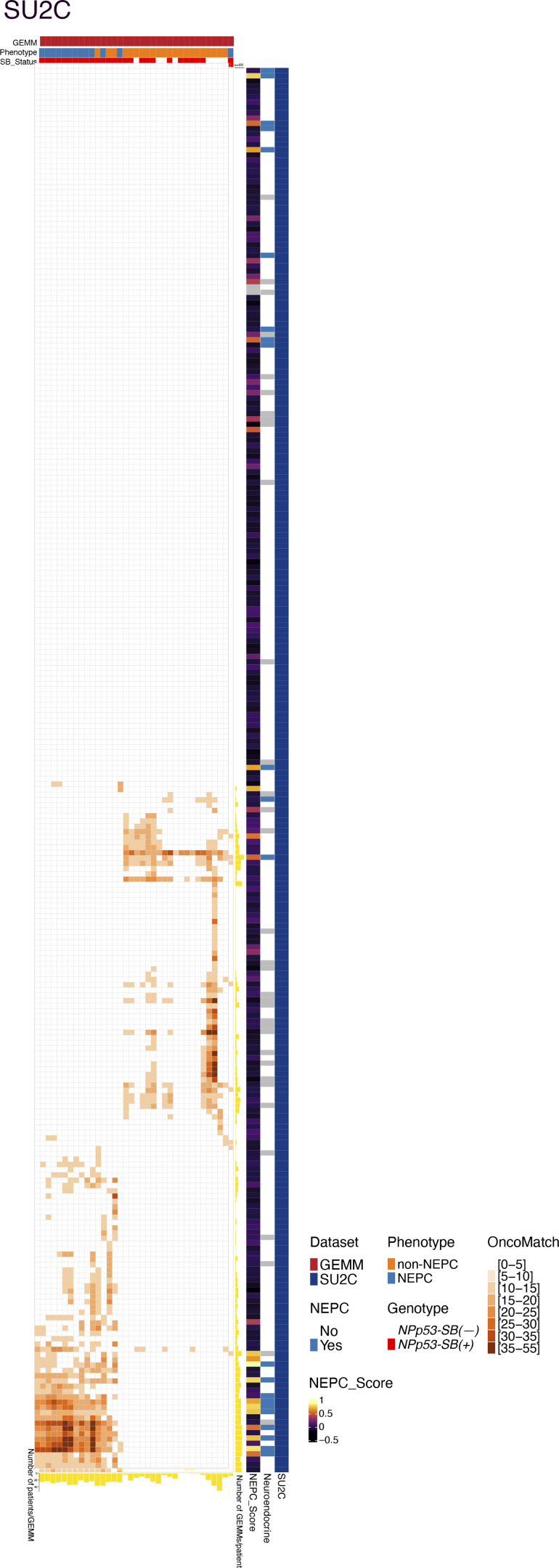
**Additional OncoMatch analyses (related to Fig. 4).** OncoMatch heatmap representing conservation of MRs between individual *SB* tumors (rows, *n* = 32) and patient tumors (columns, *n* = 266) from the SU2C cohort. Patient variables (i.e., assessment of neuroendocrine features and NEPC score) are shown on the right three bars, while GEMM variables (i.e., phenotype and genotype) are shown on the top vertical bars. Yellow bars at the right of the heatmaps show the number of GEMMs that match each patient, while the yellow bars on the bottom side show the number of patients that match each GEMM.

To directly assess conservation between mouse *C*_*3*_ NEPC tumors and human NEPC patients, we leveraged the OncoMatch algorithm (Vasciaveo et al., 2023) (Fig. 4, A and C; Fig. S2, and Table S4 A–D). For this analysis, we queried two well-characterized human prostate cancer cohorts that include NEPC patients: the Beltran cohort described above (*n* = 34 CRPC-adeno and *n* = 15 CRPC-NE, see above; Beltran et al., 2016) and the Stand Up to Cancer–Prostate Cancer Foundation study (SU2C) cohort consisting of posttreatment metastatic biopsies from mCRPC patients (*n* = 210 non-NEPC, *n* = 22 NEPC, *n* = 34 unclassified; Abida et al., 2019). Protein activity profiles for each human tumor were inferred using VIPER (Table S4 A–D) with a previously generated human prostate cancer interactome (Vasciaveo et al., 2023).

After deriving independent protein activity signatures for each mouse and human tumor, we assessed cross-species conservation by comparing the relative enrichment of their respective MR programs. Specifically, we used the aREA algorithm (Alvarez et al., 2016) to assess the 25 most activated (25↑) and 25 most inactivated (25↓) candidate MRs from each human tumor within the protein activity profiles of each mouse tumor (Table S4 A–D). A fixed MR set (25↑ + 25↓) was used to ensure comparability across cohorts because prior studies have shown that ∼50 MRs are sufficient to capture canalized, functionally relevant genetic alterations (Paull et al., 2021; Vasciaveo et al., 2023).

Following Vasciaveo et al. (2023), mouse tumors were classified as high-fidelity (i.e., conserved) models of human NEPC tumors if they met a stringent OncoMatch significance threshold (P ≤ 10^−5^, by Bonferroni corrected one-tail aREA test). Using this criterion, each mouse *C*_*3*_ NEPC tumor represented a high-fidelity model of at least one human NEPC tumor (Fig. 4 C and Fig. S2). Conversely, high-fidelity mouse NEPC models were identified for 66.7% and 45.5% of NEPC samples in the Beltran (Fig. 4 C) and SU2C cohorts (Fig. S2), respectively. Together, these analyses define the transcriptional programs and MRs associated with NEPC in mouse *SB* tumors and demonstrate their conservation in human NEPC.

### Identification of CIS genes enriched in NEPC

CIS refers to transposon insertion loci that are recurrent across multiple independent tumors and, therefore, are more likely to dysregulate genes causally associated with the accompanying tumor phenotypes (Ranzani et al., 2013; Sarver et al., 2012). Because transposon insertions occur randomly and at high frequency throughout the genome, statistical approaches—such as Poisson distribution–based analyses—are required to distinguish statistically significant CIS events from random background. Accordingly, to identify transposon-dysregulated genes associated with NEPC in mouse SB tumors, we performed genomic sequencing to map transposon insertion sites (>1 million total sites), followed by identification of the CIS (122 total CIS), and the assignment of CIS to nearby genes (330 genes, hereafter CIS genes) (Fig. 1, step 3).

**Figure 5.**
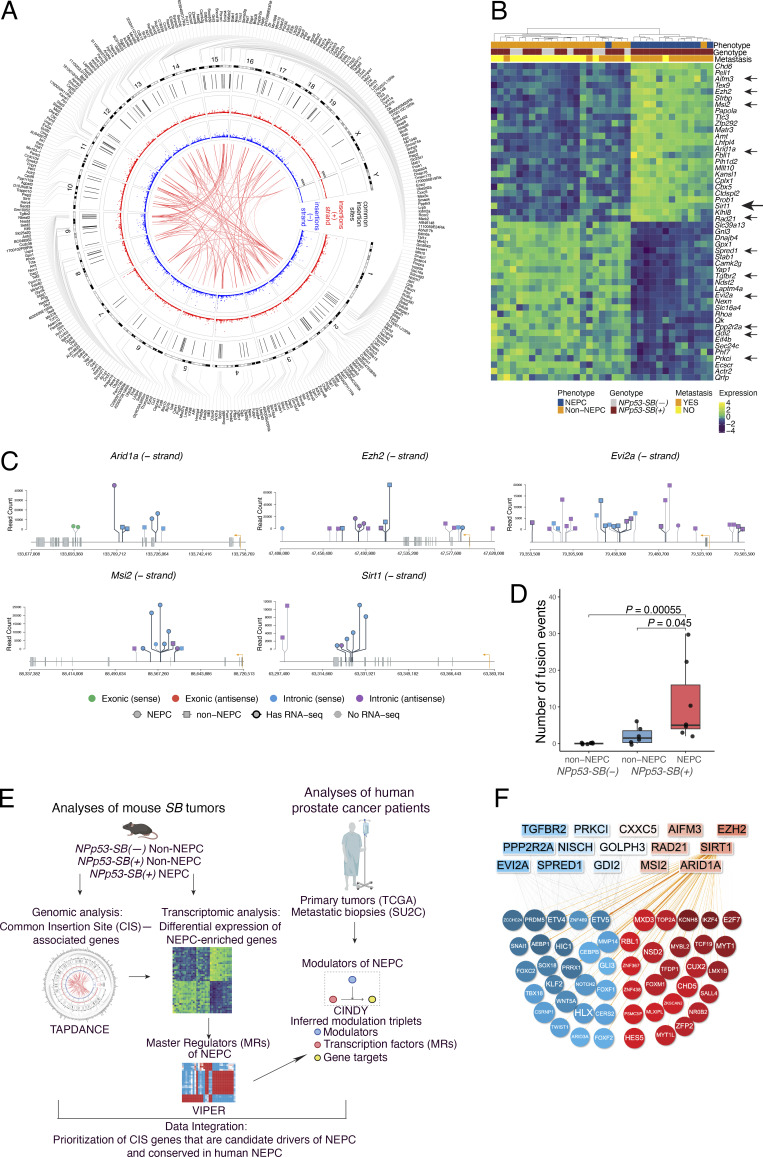
**Identification of CIS genes as candidate mechanistic determinants of NEPC. (A)** Circos plot of CIS genes identified using the TAPDANCE algorithm. Transposon insertions in the plus (red dots) and minus (blue dots) strands are individually annotated, with corresponding CIS (black lines) and the genes associated with those CIS (CIS genes, outer rim). Red lines in the center of the Circos plot connect insertions that significantly co-occur in tumors (i.e., CIS). Data are from analyses of 74 independent genomic DNA samples from the *NPp53-SB(+)* mice. **(B)** RNA-seq heatmap showing relative expression levels of the 25 most up-regulated and 25 most down-regulated CIS genes in NEPC versus non-NEPC mouse *SB* tumors. **(C)** Lollipop plots showing transposon insertion sites for selected CIS genes. TSS (orange arrows) indicate transcriptional orientation. Insertion colors indicate exonic (green/red) versus intronic (blue/purple) location and sense versus antisense strand orientation. Shape indicates NEPC (circle) versus non-NEPC (square) tumor status. Border thickness indicates samples with matched RNA-seq data. **(D)** The number of fusion events in non-NEPC and NEPC mouse *SB* tumors, as indicated. P values were calculated using the Wilcoxon rank-sum test. **(E)** CIS genes were prioritized as candidate mechanistic determinants using cross-species integration of genomic and transcriptomic data from mouse *SB* tumors with transcriptomic data from human patient cohorts as described in the text. **(F)** Visual representation of the top 15 CIS genes that are candidate modulators of NEPC (top) and the MRs they are inferred to regulate NEPC (bottom). See also Figs. S3 and S4; and Tables 2, S6, and S7.

Specifically, we carried out multiplexed Illumina sequencing of *NPp53-SB(+)* tumors (*n* = 74), using unique barcodes for each tumor sample (Table S5; see Materials and methods). Statistically significant CIS were identified with the TAPDANCE software (Sarver et al., 2012) and visualized using a Circos plot (Fig. 5 A and Table S6). This analysis identified 122 CIS distributed across multiple chromosomes on both DNA strands (Fig. 5 A and Table S6). We further used TAPDANCE to identify genes within 20 kb of each CIS. One CIS lacked a nearby gene, while the remaining 121 CIS mapped to 330 CIS genes, with most CIS associated with multiple genes; these relationships are illustrated in the outer ring of the Circos plot (Fig. 5 A and Table S6).

**Figure S3.**
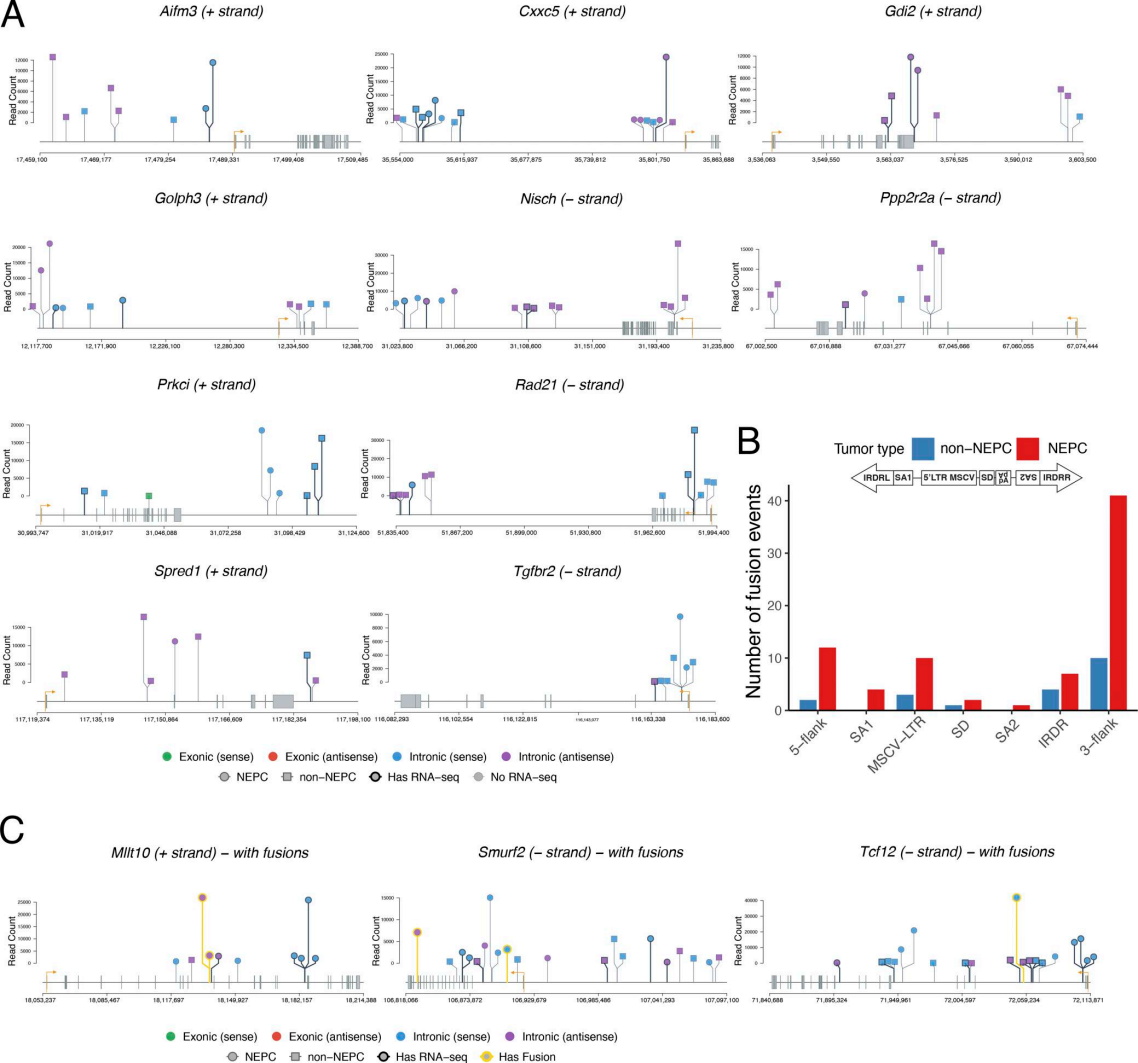
**Lollipop representation of selected CIS genes (related to Fig. 5).** (**A and C)** Lollipop plots showing transposon insertion sites for prioritized CIS genes. TSS (orange) arrows indicate transcriptional orientation. Insertion colors indicate exonic (green/red) versus intronic (blue/purple) location and sense versus antisense strand orientation. Shape indicates NEPC (circle) versus non-NEPC (square) tumor status. Border thickness indicates samples with matched RNA-seq data. Yellow highlighted colors indicate presence of transposon-RNA fusion event in that sample, at that site. **(B)** Fusion events by transposon region. Bar plot showing distribution of fusion junctions across T2/Onc2 functional regions as depicted (5′ to 3′: 5-flank, SA1, MSCV-LTR, SD, SA2, IRDR, 3-flank), stratified by tumor type. SA1 and SA2, splice acceptor sites from carp *β-actin* gene and from mouse *Engrailed2* gene; MSCV-LTR, 5′ long terminal repeat of the murine stem cell virus; SD, splice donor site; IRDR, inverted repeat/direct repeat.

**Figure S4.**
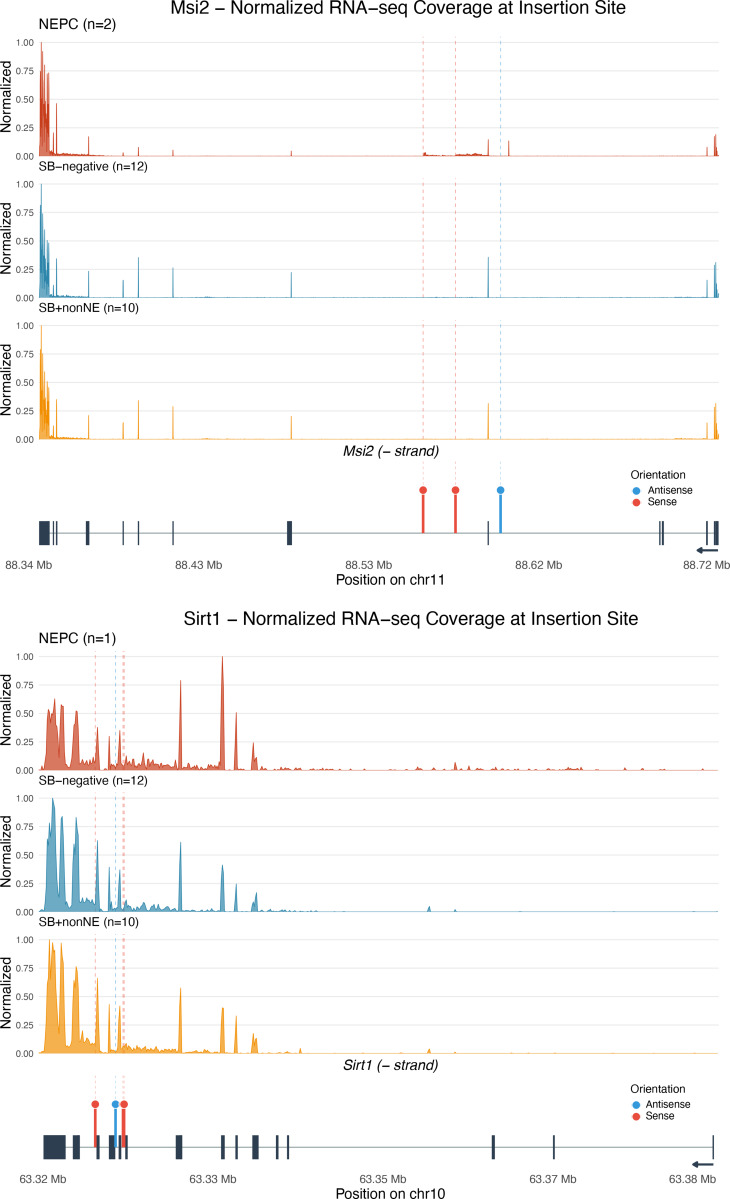
**Additional analyses of selected CIS-associated genes (related to Fig. 5).** Normalized RNA-seq coverage across the *Msi2* (top) and *Sirt1* (bottom) gene body comparing the NEPC sample with transposon insertion (*n* = 2 for *Msi2* and *n* = 1 for *Sirt1*) to SB-negative (*n* = 12) and SB-positive-non-NEPC (*n* = 10) controls. Coverage is normalized to maximum within each group. For both *Sirt1* and *Msi2*, coverage patterns may reflect transcriptional effects of insertion, though interpretation is limited by small sample sizes. Bottom lollipop plots reflect location and sense of insertions in NEPC sample(s) analyzed.

Because TAPDANCE provides precise CIS coordinates and insertion directionality (Sarver et al., 2012), we were able to analyze CIS strandedness and map the insertion sites relative to associated genes (Fig. 5, A–D; Fig. S3, A–C; Fig. S4, and Table S6). CIS insertion patterns at individual genes were visualized using lollipop plots, which depict both strandedness and location relative to transcription start sites (TSS), exons, introns, and putative upstream or downstream regulatory regions (Fig. 5 C; and Fig. S3, A and C).

As expected, given the random nature of transposition (Collier et al., 2005; Dupuy et al., 2005; Dupuy et al., 2009), CIS insertions were distributed across both the positive and negative strands and throughout the gene bodies at positions predicted to affect gene activity and RNA expression (Fig. 5, C and D; Fig. S3, A–C; Fig. S4, and Table S6). These included insertions at or near TSS (e.g., *Evi2a*, *Tgfbr2*, and *Nisch*), within or adjacent to exons (e.g., *Sirt1*, *Arid1a*, and *Pp2r2a*), within introns (e.g., *Arid1a*, *Ezh2*, and *Msi2*), and within putative 5′ or 3′ regulatory regions (e.g., *Aifm3*) (Fig. 5 C; and Fig. S3, A and C).

Additionally, in a subset of tumors for which both the genomic and RNA-seq data were available (e.g., *Sirt1* and *Msi2*), the locations of CIS insertions were consistent with observed differences in their RNA transcript (Fig. S4), consistent with an impact on gene expression. Interesting, some insertions (e.g., *Smurf2* and *Tcf12*) were predicted to generate gene fusions or alter isoform usage, potentially affecting gene function (Fig. S3 C and Table S6). Notably, CIS fusion events were more frequent in NEPC than in non-NEPC tumors (P = 0.045; Fig. 5 D and Fig. S3 B), suggesting selective enrichment of CIS in NEPC. Consistent with this, RNA-seq analysis comparing NEPC and non-NEPC *SB* tumors (Fig. 3) identified 75 CIS genes significantly enriched among differentially expressed genes (Fig. 5 B and Table S7). These NEPC-associated CIS genes were, therefore, prioritized for further analyses.

### Prioritization of CIS genes as potential mechanistic determinants of NEPC

Among the 75 NEPC-associated CIS genes, we sought to identify those most likely to represent mechanistic determinants of NEPC and to be conserved in human prostate cancer (Fig. 1, step 4). Based on prior work showing that the causal events shaping tumor cells often act as upstream modulators of MR activity (Chen et al., 2014; Paull et al., 2021), we reasoned that CIS genes with causal roles in NEPC would preferentially function as upstream modulators of NEPC MRs (Fig. 5, E and F). We further hypothesized that conserved drivers could be identified through cross-species integration of genomic and transcriptomic data from mouse *SB* tumors with transcriptomic data from human prostate cancer cohorts (Fig. 5 E).

To identify CIS genes likely to function as upstream modulators of NEPC regulatory programs, we applied the CINDy algorithm (Giorgi et al., 2014), an updated implementation of Modulator Inference by Network Dynamics (Wang et al., 2009a). CINDy assesses the statistical significance of the conditional mutual information, I[MR;T|M], where *MR* denotes a candidate MR, *T* represents its transcriptional targets (regulon), and *M* is a candidate modulator protein (Fig. 5 E). CINDy analysis was performed on two complementary human patient cohorts, namely (1) the Cancer Genome Atlas (TCGA) cohort, comprising primary, treatment naïve prostate tumors (*N* = 498; Cancer Genome Atlas Research, 2015), and (2) the SU2C mCRPC cohort described above (*N* = 266; Abida et al., 2019). For cross-species integration, we interpreted these results in the context of the MR programs inferred from mouse NEPC and non-NEPC *SB* tumors (Fig. 4 B). Specifically, we filtered CINDy modulator–MR relationships to those in which (1) the candidate modulator (*M*) corresponded to one of the 75 NEPC-enriched CIS genes (human orthologs), (2) the candidate MR was a transcription factor with significant differential VIPER protein activity between mouse NEPC and non-NEPC *SB* tumors (Fig. 4 B), and (3) the CINDy P value was <0.05. CIS genes meeting all three criteria were classified as candidate upstream modulators of the NEPC master regulatory program (Table S7).

To prioritize these candidates, we applied Fisher’s method to integrate three independent lines of evidence: (1) CIS significance as assessed by TAPDANCE (Table S6), (2) differential expression between NEPC and non-NEPC tumors (Fig. 5 B and Table S7), and (3) CINDy-inferred significance as upstream modulators of NEPC MRs (Table 2 and Table S7). Candidates were ranked by their VIPER-inferred differential protein activity between NEPC and non-NEPC tumors (Fig. 5, E and F; and, Table 2, and Table S7), reflecting their predicted functional relevance to NEPC.

**Table 2.** Prioritization of CIS genes that are candidate mechanistic determinants of NEPC

Gene	Name	Function	Total # of tumors with CIS	% of tumors with CIS (by phenotype)		CIS FDR	DGE logFC	DGE FDR	VIPER FDR	CINDy FDR	Fisher’s P value	VIPER score
				NEPC	Non-NEPC							
*Ezh2*	*Enhancer of zeste homolog 2*	Histone methyltransferase	16	38%	62%	5.15E-19	1.363	2.57E-06	5.34E-28	2.225E-308	5.00E-48	10.907
*Sirt1*	*Sirtuin 1*	NAD-dependent deacetylase	6	67%	33%	3.41E-08	0.834	1.07E-04	4.20E-18	6.434E-140	3.47E-26	8.594
*Arid1a*	*AT-Rich Interaction Domain 1A*	Chromatin remodeler	8	50%	50%	6.68E-14	0.747	5.80E-05	1.40E-12	2.225E-308	1.27E-26	6.987
*Aifm3*	*Apoptosis Inducing Factor Mitochondria Associated 3*	Apoptotic regulator, mitochondria	8	38%	62%	8.51E-14	3.733	7.40E-06	5.78E-11	2.225E-308	8.04E-26	6.445
*Rad21*	*Double-Strand-Break Repair Protein Rad21 Homolog*	DNA repair	8	17%	83%	1.21E-10	0.697	4.56E-04	5.93E-10	5.055E-124	4.57E-20	6.082
*Msi2*	*Musashi RNA Binding protein 2*	Cell cycle regulation	10	80%	20%	1.56E-12	1.727	7.71E-06	1.76E-09	1.3778E-80	3.84E-23	5.905
*Cxxc5*	*CXXC Finger Protein 5*	DNA damage, WNT signaling	15	47%	53%	6.81E-14	0.860	5.35E-04	9.90E-02	2.225E-308	3.06E-15	1.287
*Golph3*	*Golgi Phosphoprotein 3*	Golgi trafficking	11	36%	64%	2.29E-04	−0.520	7.31E-04	9.56E-02	2.340E-304	2.89E-06	−1.307
*Gdi2*	*GDP Dissociation Inhibitor 2*	Vesicular trafficking	8	25%	75%	4.96E-11	−0.627	1.27E-05	6.76E-07	2.225E-308	5.38E-19	−4.832
*Prkci*	*Protein Kinase C Iota*	Cytoskeleton, cell migration	8	25%	75%	7.38E-06	−0.813	1.84E-04	1.63E-07	2.225E-308	1.52E-13	−5.108
*Nisch*	*Nischarin*	Cell migration	13	31%	69%	6.95E-13	−0.632	4.49E-04	3.83E-10	2.225E-308	2.03E-22	−6.152
*Spred1*	*Sprouty Related EVH1 Domain Containing 1*	Activation of MAPK	7	29%	71%	4.49E-05	−2.043	3.75E-07	4.57E-27	2.225E-308	2.88E-34	−10.710
*Tgfbr2*	*Transforming Growth Factor Beta Receptor 2*	Cell proliferation	7	29%	71%	8.99E-13	−2.219	4.64E-05	4.19E-27	3.610E-249	8.65E-40	−10.718
*Ppp2r2a*	*Protein Phosphatase 2 Regulatory Subunit B alpha*	Cell growth and division	9	11%	89%	4.53E-14	−1.233	1.78E-06	3.84E-27	2.225E-308	1.73E-42	−10.726
*Evi2a*	*Ecotropic Viral Integration Site 2A*	Cell surface receptor	19	11%	89%	1.99E-42	−2.250	1.89E-04	1.09E-38	2.225E-308	7.63E-80	−12.956

CIS FDR: adjusted P value using false discovery rate for each common-insertion site from TAPDANCE analysis. DGE logFC: logFold-change between NEPC versus non-NEPC tumors from differential gene expression analysis. DGE FDR: adjusted P value using false discovery rate for differential gene expression between NEPC versus non-NEPC tumors. VIPER FDR: adjusted P value using false discovery rate for MRs from VIPER analysis. CINDy FDR: adjusted P value using false discovery rate for each candidate modulator gene from CINDy analysis. VIPER score: VIPER score from differential protein activity analysis between NEPC versus non-NEPC tumors.

### SIRT1 promotes NEPC in human prostate cancer

**Figure 6.**
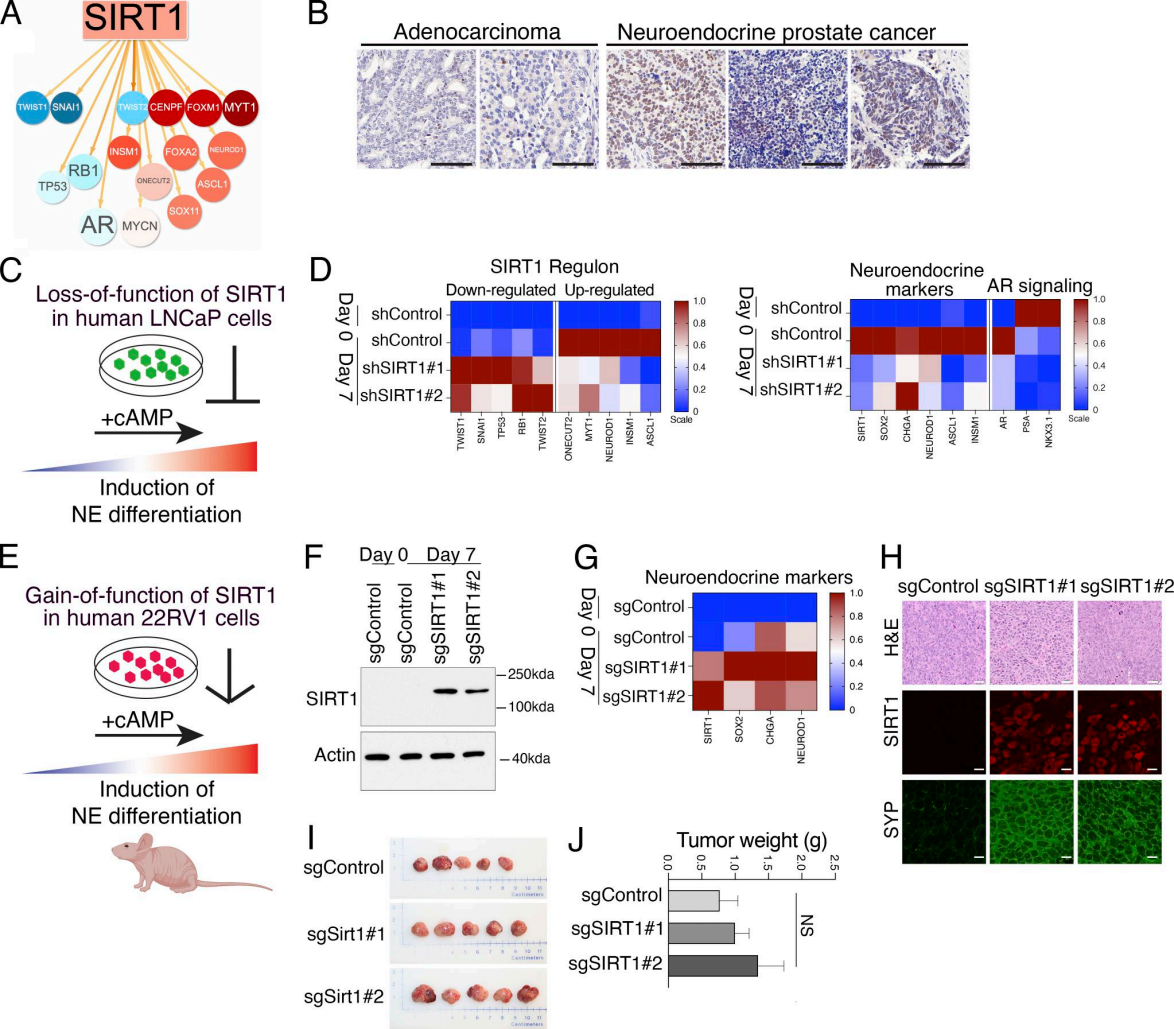
**SIRT1 promotes NEPC in human prostate cancer cell models. (A)** Visual depiction of a subset of the SIRT1 regulon showing NEPC MRs it is predicted to up-regulate (red) or down-regulate (blue) in prostate cancer. **(B)** Expression of SIRT1 protein in human prostate cancer. IHC for SIRT1 was performed on seven independent patient tumors (see Materials and methods). Scale bars represent 100 µm. **(C and D)**. Loss-of-function of *SIRT1* in LNCaP cells. **(C)** Schematic diagram showing that LNCaP cells can be induced toward neuroendocrine differentiation by treatment with db-cAMP and IBMX (abbreviated +cAMP). shRNA silencing *SIRT1* is predicted to inhibit this transition. **(D)** Heatmap representation of relative expression levels of the SIRT1 regulon (left) and known markers of NEPC or androgen signaling (right). Data show the results of real-time PCR analyses from day 0 (before NEPC induction) and day 7 (7 days after induction) for the shControl and two independent shRNA for SIRT1. Experiments were done with a minimum of five independent RNA samples for each condition. **(E–H)** Gain-of-function of *SIRT1* in 22RV1 cells. **(E)** Schematic representation showing treatment of 22RV1 cells with +cAMP for induction toward neuroendocrine differentiation, as in panel C. *SIRT1* expression was induced in 22RV1 cells using with CRISPRa and analyzed in vitro (F and G) and in vivo (H–J). **(F)** Western blot showing protein expression levels of SIRT1 in the 22RV1 cells. Actin, control for protein loading; positions of molecular weight markers are shown. The experiments were repeated a minimum of three times with three biological replicates in each. **(G)** Heatmap representation of relative gene expression levels of known markers of NEPC. **(H–J)** Results of 22RV1 cells grown orthotopically in host nude mice (*n* = 5/group). **(H)** Representative images of the 22RV1 prostate tumors. Shown are H&E staining, immunofluorescence (IF) staining for SIRT1 and synaptophysin (SYP). Scale bars represent 20 µm for H&E, and 10 µm for IF images. **(I)** Prostate tumors collected at the time of sacrifice. **(J)** Summary of tumor weights. See also Fig. S5. Source data are available for this figure: SourceData F6.

**Figure 7.**
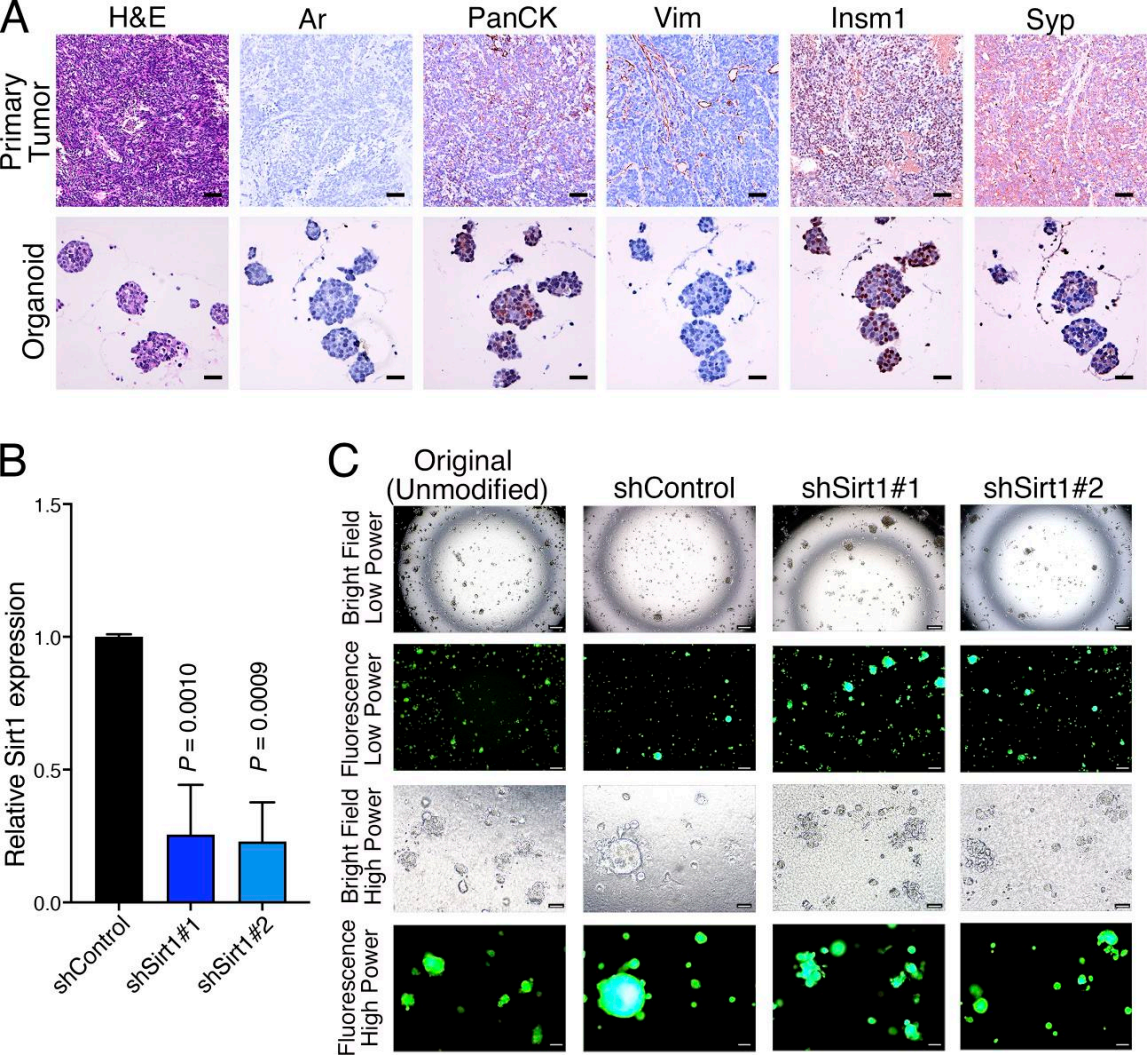
**Establishment of an organoid model of NEPC from *NPp53* mice. (A)** Representative images of the parental tumor and the corresponding organoid lines derived from an *NPp53* mouse and propagated in vitro. The images show H&E or immunostaining for the indicated markers. Scale bars represent 50 μm for the tumors and 20 μm for the organoids. **(B)** Real-time PCR showing relative expression of *Sirt1* in the organoid line at baseline (shControl) and following silencing with two independent shRNA (shSirt1#1 or shSirt1#2). Data are normalized using *Gapdh* as internal control. The experiments were repeated three times with two biological replicates. **(C)** Representative bright-field and fluorescence images of *NPp53* organoids as indicated. Scale bars represent 200 μm for low power and 50 μm for high power.

**Figure 8.**
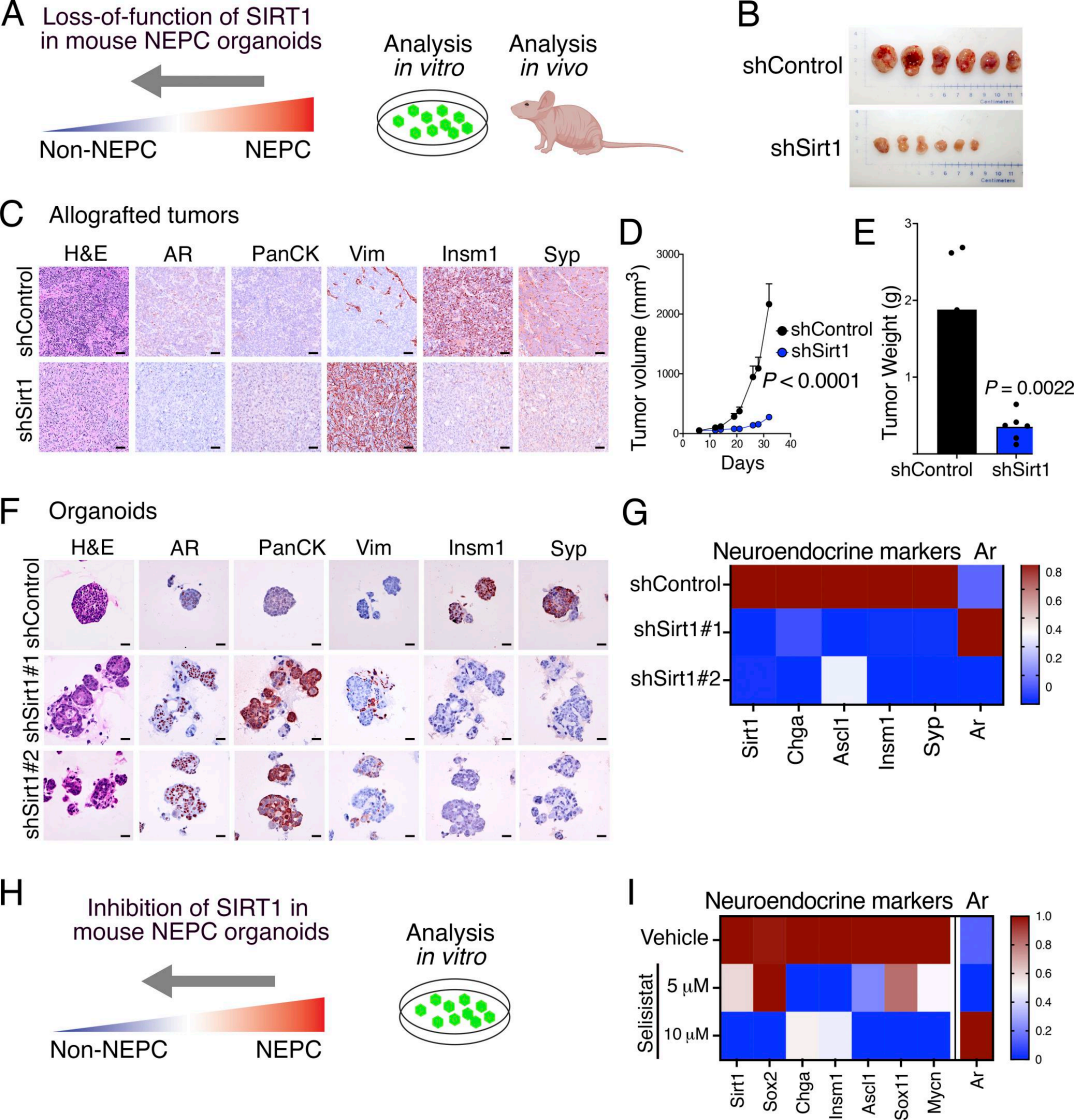
**SIRT1 silencing or pharmacological inhibition reverts NEPC. (A–G)** *Sirt1* silencing in a mouse organoid model of NEPC. **(A)** Schematic representation showing mouse NEPC organoids treated with shRNAs targeting *Sirt1* and subjected to in vitro and in vivo analyses. **(B)** Shown are images of allografted tumors from the indicated groups imaged at the time of euthanasia (*n* = 6/group). **(C)** Representative images of allografted tumors grown from organoids (*n* = 6/group). Shown are H&E and immunostaining for AR, PanCK, Vimentin (Vim), Insm1, and synaptophysin (Syp). Scale bar = 50 μm. **(D)** Tumor volume measurements for the indicated groups. *N* = 6/group. P value was calculated using a two-way ANOVA test. **(E)** Bar plot with overlaid dots showing the tumor weight (g) for the indicated groups of mice (*n* = 6/group). P value was calculated using the Welch’s two sample *t* test. **(F)** Representative images of organoids from the indicated groups (*n* = 6/group). Shown are H&E and immunostaining for the indicated markers. Scale bar = 20 μm. **(G)** Heatmap representation of relative expression levels of the *Sirt1*, *Ar*, and the indicated markers of NEPC. **(H and I)** Pharmacological inhibition of Sirt1 in mouse NEPC organoids. **(H)** Schematic representation showing mouse NEPC organoids treated with the SIRT1 inhibitor Selisistat. **(I)** Heatmap representation of relative expression levels of *Sirt1*, *Ar*, and the indicated markers of NEPC for the indicated groups of organoids. Experiments were performed in quadruplicate and with a minimum of two independent biological replicates. See also Fig. S5.

**Figure S5.**
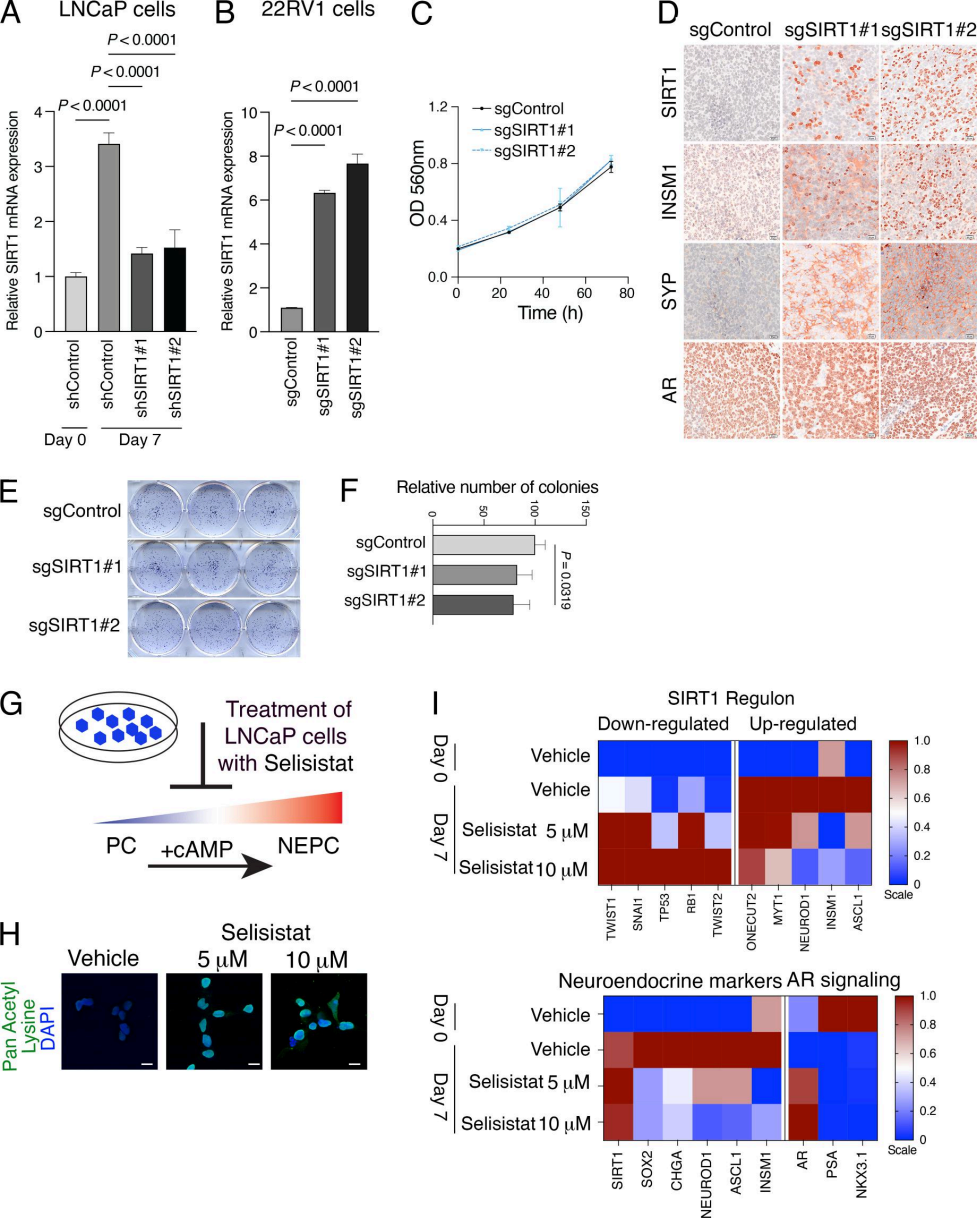
**Additional analyses of validation studies in human cells (related to Figs. 6 and 8** **).** **(A and B)** Real-time PCR showing relative expression of *SIRT1* in human prostate cancer cell lines. Data are normalized using *GAPDH* as internal control. **(A)** LNCaP cells with control shRNA or silenced for *SIRT1* with two independent shRNA (shSIRT1#1 or shSIRT1#2). **(B)** 22RV1 CRISPRa cell line with sgControl or two independent sgs to activate *SIRT1* (sgSIRT1#1 or sgSIRT1#2). **(C–E)** Analyses of activation of SIRT1 in 22RV1 CRISPRa cells in vitro (C and E) and in vivo (D). **(C)** Quantification of cell proliferation from 0 to 72 h of growth measured by detection of MTT absorbance. OD, optical density. **(D)** Representative images of 22RV1 prostate tumors (*n* = 6/group). Shown is immunostaining for SIRT1, INSM1, synaptophysin (SYP), and AR. Scale bars represent 20 µm. **(E and F)** Crystal violet staining of colony formation assay showing 22RV1 CRISPRa cell lines treated for 12 days with sgControl, sgSIRT1 #1 or #2 (E), and quantification of the number of colonies (F). **(G–I)** Pharmacological inhibition of SIRT1 in LNCaP cells. **(G)** Strategy for induction of NEPC in LNCaP cells by treatment with db-cAMP and IBMX (abbreviated +cAMP). Treatment with the SIRT1 inhibitor Selisistat is predicted to inhibit this transition. **(H)** Relative levels of lysine acetylation following treatment of LNCaP cells with 5 or 10 µM Selisistat. Scale bars represent 20 µm. **(I)** Heatmap representation of relative expression levels of the SIRT1 regulon (top) and known markers of NEPC or androgen signaling (bottom). Experiments were done in triplicate with two independent biological replicates.

Among the prioritized candidates, we focused on the NAD-dependent deacetylase *Sirt1* (Fig. 1, step 5). Sirt1 exhibited strong VIPER-inferred differential protein activity in NEPC versus non-NEPC tumors (VIPER score, 8.59, VIPER FDR, 4.2 X 10^−18^; Table 2), and a high percentage of CIS events in NEPC tumors (67%; Table 2). RNA-seq coverage analysis also suggested differences in the *Sirt1* transcript between NEPC and non-NEPC tumors, although interpretation was limited by the small sample size (Fig. S4). Further, the predicted regulon of SIRT1 includes several established NEPC regulators, including MYT1, NEUROD1, INSM1, and ASCL1 (Fig. 6 A). Consistent with these findings, SIRT1 was highly expressed (>70% positive cells) in patient samples with neuroendocrine pathology but showed minimal expression (<5%) in non-NEPC tumors (Fig. 6 B). Based on this convergent evidence, we evaluated the functional relevance of *SIRT1* using gain- and loss-of-function approaches in human prostate cancer cell lines induced toward NEPC and in a mouse NEPC organoid model (Figs. 6, 7, 8, and S5).

First, we examined the effects of *SIRT1* loss-of-function in Lymph Node Carcinoma of the Prostate (LNCaP) cells (Horoszewicz et al., 1980), an AR-dependent prostate cancer cell line that can be induced to differentiate to a neuroendocrine-like phenotype when deprived of androgens and treated with agents that increase the intracellular levels of cyclic AMP (hereafter denoted +cAMP; Fig. 6 C) (Bang et al., 1994; Burchardt et al., 1999; Shen et al., 1997). *SIRT1* is expressed at low levels prior to NEPC induction (day 0) but was significantly increased following induction (day 7). This induction-associated increase in *SIRT1* expression was effectively suppressed by two independent *SIRT1*-directed shRNAs (Fig. S5 A).

In the control LNCaP cells, NEPC induction resulted in increased expression of genes comprising the SIRT1 regulon that are predicted to be activated by SIRT1 (*ONECUT2*, *MYT1*, *NEUROD1*, *INSM1*, and *ASCL1*), and a corresponding decrease in expression of those predicted to be down-regulated by SIRT1 (*TWIST1*, *SNAI1*, *TP53*, *RB1*, and *TWIST2*) (Fig. 6 D). However, this change in gene expression was abrogated by silencing *SIRT1* (Fig. 6 D). Furthermore, NEPC induction of the LNCaP cells (day 7) resulted in increased expression of NEPC markers (*SOX2*, *CHGA*, *NEUROD1*, *ASCL1*, and *INSM1*), which was abrogated by depletion of *SIRT1* (Fig. 6 D), supporting the idea that *SIRT1* supports induction of NEPC differentiation of LNCaP cells.

To extend these findings, we performed gain-of-function studies using a human prostate cancer cell line, 22RV1 (Sramkoski et al., 1999), which exhibits low* SIRT1* expression in both untreated cells (day 0) and following NEPC induction with +cAMP (day 7) (Fig. 6, E and F; and Fig. S5 B). We, therefore, investigated whether enforced *SIRT1* expression promotes NEPC differentiation in vitro and in vivo following orthotopic implantation (Fig. 6, E–J; and Fig. S5, B–F). SIRT1 was activated in 22RV1 cells using a previously described CRISPR activation (CRISPRa) system (Arriaga et al., 2024). Cells expressing either a control sgRNA or two independent *SIRT1*-targeting sgRNAs were generated, both of which produced robust increases in SIRT1 mRNA and protein levels (Fig. 6, F and G; and Fig. S5 B). Although *SIRT1* activation had modest or negligible effects on cell proliferation and colony formation (Fig. S5, C, E, and F), it induced a marked up-regulation of key NEPC markers, including *SOX2*, *CHGA*, and *NEUROD1* (Fig. 6 G).

Consistent with these in vitro findings, orthotopic implantation of 22Rv1 CRISPRa cells into the prostates of male host mice revealed only modest effects on tumor growth (Fig. 6, I and J), but a striking increase in expression of NEPC markers, including SYP and INSM1, with minimal effects on AR expression (Fig. 6 H and Fig. S5 D). Together, these results demonstrate that gain-of-function of SIRT1 promotes NEPC differentiation in human prostate cancer models.

To more directly assess the role of *Sirt1* in NEPC, we developed a mouse organoid model with an overt NEPC phenotype (Fig. 7). This organoid was derived from a baseline (i.e., non-*SB*) *NPp53* mouse that developed a rare spontaneous NEPC tumor, as defined by characteristic histopathological features, low expression of AR, pan-CK, and vimentin, and high expression of the neuroendocrine markers Insm1 and Syp (Fig. 7 A). The derived organoid faithfully recapitulated the parental tumor phenotype, retaining low AR, pan-CK, and vimentin expression and high Insm1 and Syp expression (Fig. 7 A). Notably, *Sirt1 *expression was high in this NEPC organoid model (Fig. 7 B).

To directly assess the functional role of *Sirt1* in maintaining the NEPC phenotype, we silenced its expression in the NEPC organoid model using an inducible shRNA and evaluated the effects on NEPC differentiation and tumor growth both in vitro and in vivo (Fig. 7, B and C; and Fig. 8, A–G). *Sirt1* silencing resulted in a striking reversal of neuroendocrine features, evident by increased expression of AR, pan-CK, and vimentin, accompanied by reduced expression of the neuroendocrine markers Insm1 and Syp, both in vitro and following subcutaneous growth in vivo (Fig. 8, C and F). This phenotypic reversion was associated with a profound reduction in tumor growth in vivo (Fig. 8, D and E). These findings demonstrate that *Sirt1* is required for maintenance of the NEPC state and that its suppression is sufficient to revert the NEPC phenotype and inhibit NEPC tumor growth.

Given that Sirt1 depletion both prevented NEPC induction and reversed established NEPC, we next asked whether pharmacological inhibition of SIRT1 could phenocopy these effects in human prostate cancer cells and mouse NEPC organoids (Fig. 8, H and I; and Fig. S5, G–I). For these studies, we used the SIRT1-specific inhibitor selisistat, which has been shown to inhibit SIRT1 lysine deacetylase activity in multiple cellular contexts (Adams et al., 2024; Napper et al., 2005; Westerberg et al., 2015). In our models, selisistat effectively inhibited lysine deacetylase activity at 5–10 μM without compromising overall cell viability (Fig. S5 H), consistent with doses used previously (Adams et al., 2024).

Pharmacological inhibition of SIRT1 abrogated NEPC induction in LNCaP cells and reversed the NEPC phenotype in the mouse organoid model (Fig. 8, H and I; and Fig. S5, G–I). This was reflected by altered expression of SIRT1-regulated targets, including *ONECUT2* and *MYT1* (Fig. S 5 I), as well as decreased expression of neuroendocrine markers such as *SOX2,INSM1*, and *ASCL1* (Fig. 8 I and Fig. S5 I). Collectively, these findings demonstrate that SIRT1 promotes NEPC differentiation, while its genetic or pharmacological inhibition suppresses or reverses the NEPC phenotype.

## Discussion

Forward genetic screens, particularly those based on *SB* mutagenesis, have proven powerful for unbiased identification of cancer drivers and therapeutic targets (Beckmann and Largaespada, 2020; Copeland and Jenkins, 2010). Because *SB* mutagenesis is implemented in autochthonous mouse models, it enables the discovery of genetic events as they arise somatically during disease progression within the native tumor microenvironment, closely mirroring the evolutionary processes that occur in human cancers. In this study, we employed a *SB* mutagenesis screen to identify molecular drivers of NEPC, an aggressive prostate cancer variant that is increasing in incidence as a consequence of therapy resistance (Abida et al., 2019; Watson et al., 2015). By integrating tumor phenotypic analyses with transcriptomic profiling and genomic mapping of transposon insertion sites in a cross-species framework, we identified the NAD-dependent deacetylase SIRT1 as a targetable driver of NEPC.

Unlike prior *SB* mutagenesis screens in prostate cancer mouse models, which were based on loss of the *Pten* tumor suppressor gene alone (Ahmad et al., 2016; Rahrmann et al., 2009), our study employed a model with concurrent loss of *Pten* and *Trp53* (*NPp53* mice), which is predisposed to develop aggressive disease (Zou et al., 2017). Using the *NPp53* model as the basis for the screen enabled identification of drivers of advanced prostate cancer and, in particular, facilitated interrogation of the molecular determinants of NEPC, a highly lethal subtype that has emerged in the context of treatment resistance (Beltran et al., 2019; Watson et al., 2015). Notably, NEPC phenotypes in *NPp53-SB(+)* mice arise in the setting of intact androgen signaling and in the absence of therapeutic intervention, providing a unique opportunity to investigate the *de novo* cellular events that give rise to lethal prostate cancer.

A major limitation of *SB* and other forward genetic screens is the challenge of prioritizing functional drivers from large candidate gene sets. Our integrative framework addresses this limitation by combining *SB*–derived genomic and transcriptomic data with regulatory network–based analyses across both mouse and human prostate cancer, enabling systematic identification of CIS genes most likely to control disease-relevant transcriptional programs. Specifically, we integrate phenotypic, genomic, and transcriptomic analyses of mouse *SB* tumors with human prostate cancer cohorts to identify and prioritize CIS genes with mechanistic roles in driving NEPC. This work builds on our prior studies demonstrating that cross-species reverse engineering of transcriptomic data can uncover gene regulatory networks and nominate modulators that influence transcription factor activity, thereby revealing key determinants of cancer (Aytes et al., 2014; Giorgi et al., 2014; Paull et al., 2021; Vasciaveo et al., 2023).

Although this study focuses on *SIRT1*, our screen identified several additional candidate CIS genes of interest. Some have been previously implicated in NEPC, particularly the lysine methyltransferase *Ezh2* (Enhancer of Zeste 2, PRC2 subunit) (Dardenne et al., 2016; Ku et al., 2017). Others, such as the TGFβ type II receptor (*Tgfbr2*), have not been directly linked to NEPC but are components of signaling pathways—here, TGFβ—known to regulate lineage plasticity and prostate cancer progression (Hao et al., 2018). Additional candidates, including the RNA-binding protein *Msi2*, have not been previously associated with NEPC but play established roles in cancer and stem cell maintenance (Kharas et al., 2010). Notably, although *Evi2a* resides together with *Evi2b* and *Omg* within the *Nf1* tumor suppressor locus (O'Connell et al., 1990) —a frequent target of *SB* mutagenesis—*Evi2a* was the only gene in this region to pass all prioritization criteria.

*SIRT1* is a member of the sirtuin family, originally identified in non-mammalian systems for their roles in lifespan regulation (Haigis and Sinclair, 2010), and encodes an NAD-dependent lysine deacetylase that promotes heterochromatin formation (Vaquero et al., 2004). Beyond its canonical epigenetic functions, *SIRT1* plays key roles in the nervous system and has been implicated in neurodegenerative and neuroendocrine diseases (Fujita and Yamashita, 2018). *SIRT1* also functions in cancer, in part through interactions with p53 (Lin and Fang, 2013). Notably, *SIRT1* was first identified in yeast as a regulator of the SWI/SNF chromatin remodeling complex (Mittal and Roberts, 2020), raising the possibility that its role in NEPC may be mediated through modulation of SWI/SNF activity—particularly given the established importance of this complex in prostate cancer and lineage plasticity (Cyrta et al., 2020; Thienger et al., 2026).

The role of *SIRT1* in cancer is highly context dependent, with evidence supporting both oncogenic or tumor-suppressive functions depending on cellular state and disease stage (Lin and Fang, 2013). Prior studies have reported tumor-suppressive effects based on preinvasive phenotypes following *SIRT1* loss, as well as tumor-promoting roles in advanced disease (Byles et al., 2012; Di Sante et al., 2015; Huang et al., 2021; Huang et al., 2022; Powell et al., 2011). This functional duality underscores the need to define the stage- and context-specific roles of SIRT1 during tumor progression.

Although *SIRT1* is up-regulated in prostate cancer, including in NEPC (Huang et al., 2021; Huffman et al., 2007; Natani et al., 2021; Ruan et al., 2018), its functional significance in this setting has remained unclear. Our findings reconcile these observations by demonstrating that *SIRT1* plays a pivotal role in promoting NEPC, revealing a context-dependent function that extends beyond general tumor growth to the regulation of lineage plasticity and neuroendocrine differentiation. Notably, while our studies establish a role for *SIRT1* in NEPC, the *SB* data analysis does not prove that the insertion events lead to gain of function of *Sirt1*.

Importantly, our data suggest that targeting *SIRT1* may suppress or reverse progression toward NEPC. Pharmacologic SIRT1 inhibitors, such as selisistat, are Food and Drug Administration–approved and have been evaluated in Phase I trials (Westerberg et al., 2015), highlighting SIRT1 as an attractive and clinically actionable target for lethal prostate cancer that warrants further investigation in future clinical studies.

## Materials and methods

### Generation and analysis of *SB* mice

All experiments using animals were performed according to protocols approved by the Institutional Animal Care and Use Committee at Columbia University Irving Medical Center. The mice used in this study are on a mixed strain (C57BL/6J;129S2) background and were housed in pathogen-free barrier conditions under 12-h light/dark cycles and with temperature and humidity at 20–25°C and 30–70%, respectively. Since the focus of our study is on prostate cancer, only male mice were used. The *SB* mice have a Cre*-*inducible transposase allele (*Rosa26-LSL-SB11;*Geurts et al., 2003; Starr et al., 2009) with or without the *T2/Onc2* transposon allele (National Cancer Institute [NCI] Mouse Repository, Frederick National Laboratory, strain 01XGA; Dupuy et al., 2005) such that the *SB(−)* mice have only the *Rosa26-LSL-SB11* allele, while the *SB(+)* mice have both the *Rosa26-LSL-SB11* and the *T2/Onc2* alleles. *SB(−)* and *SB(+)* mice were crossed with: (1) *NPp53* mice (Zou et al., 2017) to generate *NPp53-SB(−)* mice (*Nkx3.1*^*CreERT2/+*^*; Pten*^*flox/flox*^*; Trp53*^*flox/flox*^*; R26*^*LSL-SB11/LSL-SB11*^) and *NPp53-SB(+)* mice (*Nkx3.1*^*CreERT2*^*; Pten*^*flox/flox*^*; Trp53*^*flox/flox*^*; R26-*^*LSL-SB11/LSL-SB11*^*; T2/Onc2*); or (2) *NP* mice (Floc’h et al., 2012) to generate *NP-SB(−)* mice (*Nkx3.1*^*CreERT2*^*; Pten*^*flox/flox*^*; R26*^*LSL-SB11/LSL-SB11*^) and the *NP-SB(+)* mice (*Nkx3.1*^*CreERT2*^*; Pten*^*flox/flox*^*; R26*^*LSL-SB11/LSL-SB11*^*; T2/Onc2*). Since the *T2/Onc2* transposon is located on chromosome 1, insertions on this chromosome were disregarded in subsequent analysis to overcome complications related to local hopping (Liang et al., 2009). A summary of all mice used in this study is provided in Table S1.

Tumor induction was achieved using the *Nkx3.1*^CreERT2^ allele, which has an inducible Cre recombinase that is specifically activated in luminal prostatic epithelial cells following delivery of tamoxifen (Wang et al., 2009b). Mice were genotyped prior to tumor induction. For tumor induction, mice were administered vehicle (corn oil) or tamoxifen (100 mg/kg in corn oil) (T5648; Sigma-Aldrich) via oral gavage at 2–3 mo of age once daily for 4 consecutive days. Mice were monitored three times weekly, and euthanized when their body condition score was <1.5, or when they experienced body weight loss ≥20% or signs of distress, such as difficulty breathing or bladder obstruction. At their terminal endpoint, mice were euthanized via carbon dioxide inhalation followed by cervical dislocation and were necropsied. Harvested tissues were visualized using an Olympus SZX16 microscope. Tissues were snap-frozen in liquid nitrogen for DNA or RNA isolation, or were fixed in 10% formalin (Thermo Fisher Scientific) for H&E, immunohistochemical and immunofluorescence staining.

Histopathological scoring of the prostate tumors was assessed independently by two pathologists (S. de Brot and A. Rodriguez-Calero) based on evaluation of H&E-stained tissues, some with confirmation by immunohistochemistry for Syp and Ki67 as summarized in Table S1. As described in Ittmann et al. (2013) and Shappell et al. (2004), histomorphologic features indicative of neuroendocrine differentiation included: (1) organoid nesting trabecular growth pattern, with peripheral palisading, and rosette formation; (2) tumor cells smaller than the diameter of three small lymphocytes, having scant cytoplasm, with oval or spindled hyperchromatic nuclei; and (3) high mitotic count; and (4) frequently presence of extensive necrosis.

Immunostaining was done using 3 μm formalin-fixed sections as described (Zou et al., 2017). Briefly, sections were deparaffinized in xylene followed by heat-induced antigen retrieval using Citrate-Based Antigen Unmasking Solution at pH 7.0 (Vector Labs). Sections were blocked in 10% normal goat serum, incubated with primary antibodies overnight at 4°C, and with secondary antibodies for 1 h at room temperature. For immunohistochemistry, signal detection and visualization were performed using the Vectastain ABC system followed by NovaRed Substrate Kit (Vector Labs), counterstained with hematoxylin and mounted with Clearmount (American Master*Tech Scientific). Images were captured using an Olympus VS120 whole-slide scanning microscope. For immunofluorescence, tissues were incubated with primary and secondary antibodies as above and then stained with DAPI and mounted with Vectashield antifade mounting medium (Vector Labs). Images were captured using a Leica TCS SP5 confocal microscope. Quantification of Ki67 staining was performed manually on a minimum of 8,000 cells per group based on three independent tumors with a minimum of five sections per tumor, as described in Zou et al. (2017). All antibodies and secondary antibodies are described in Table S8.

Validation of mRNA expression levels was done by quantitative real-time PCR using the QuantiTect SYBR Green PCR kit (Qiagen) using mouse *Gapdh* as the control (Zou et al., 2017). Primer sequences are provided in Table S8.

### Transcriptomic analyses of *SB* prostate cancer mouse models

RNA-seq was performed on 35 RNA samples from 32 independent mouse prostate tumors, including 8 *NPp53-SB(−)* tumor samples and 27 *NPp53-SB(+)* tumor samples that included 13 histologically defined as NEPC and 22 histologically characterized as non-NEPC (Table S1). We also analyzed four *NP-SB(−)* and four *NP-SB(+)* tumors, all of which were histologically defined as non-NEPC (Table S1). RNA was prepared from snap-frozen tissues that were homogenized in TRIzol (Thermo Fisher Scientific) and extracted using the MagMAX-96 total RNA isolation kit (Thermo Fisher Scientific). Total RNA was enriched for mRNA using poly-A pull-down; only samples having between 200 ng and 1 µg and with an RNA integrity number >8 were used.

Libraries were made using an Illumina TruSeq RNA prep-kit version 2 or TruSeq Stranded mRNA library prep kit and sequenced using an Illumina HiSeq2500/4000 or NovaSeq6000. RNA-seq profiles were generated by mapping mRNA reads to the mouse reference genome (version GRCm38 mm10), using kallisto version 0.44.0. RNAseq raw counts were normalized and variance stabilized using DESeq2 (version 1.36.0) package (Bioconductor) in R-studio 2023.03.0+385, R version 4.2.3 (R Foundation for Statistical Computing).

For the generation of gene expression heatmaps, variance stabilized counts were z-scaled by row and visualized using the ComplexHeatmap (version 2.14.0) package (Bioconductor) in R-studio 2023.03.0+385, R version 4.2.3 (R Foundation for Statistical Computing). PCA was performed using the plotPCA function from the DESeq2 (version 1.42.1) package, using the top 500 highly variable genes computed from variance stabilized count data.

To identify genes differentially expressed in histologically defined NEPC versus non-NEPC SB mouse tumors, we used edgeR (version 4.0.16). Read counts were modeled using Genewise Negative Binomial Generalized Linear Models with Quasi-Dispersion Estimation (glmQLFit) after having estimated gene dispersion using Empirical Bayes Tagwise Dispersions for Negative Binomial GLMs (estimateGLMTagwiseDisp). The contrast matrix was generated by comparing SB(+) tumors that displayed NEPC with those that did not display NEPC. The list of statistically significantly (FDR <0.1, Benjamini-Hochberg corrected) differentially expressed genes is provided in Table S2 A; the dataset is provided in GSE271053. To compare the mouse RNA-seq data with human NEPC gene signatures, mouse genes were mapped to their human orthologs and compared with the AR, NEURO I, and NEURO II gene expression sets reported in Labrecque et al. (2019). Data were visualized as a heatmap and provided in Table S2 B.

Protein activity-based cluster analysis was done following VIPER-based analysis of RNA-seq profiles from the mouse SB tumors including the 13 histologically defined NEPC tumors and the 22 histologically defined non-NEPC tumors (see above). First, a differential gene expression signature (DGES) was computed for each sample as above (Table S2 A). Then the VIPER algorithm (Alvarez et al., 2016) was used to transform each DGES into a differential protein activity signature. A summary of VIPER protein activity is provided in Table S3 A.

Clusters were identified by the hierarchical clustering, as implemented by hclust (version 4.3.2), with Ward’s agglomeration method (Fionn and Legendre, 2014). Spearman’s correlation of the respective protein activity profiles was used as a sample-to-sample distance metric. This yielded a list of MR proteins representing candidate drivers of the cell states associated with each cluster. For visualization purposes, only the 15 most statistically significant differentially MRs are shown, as assessed by P value integration across the samples of each cluster (*n* = 3), using Stouffer’s method. A summary of protein activity for each cluster is provided in Table S3 B.

### OncoMatch protein activity-based analysis of mouse and human tumors

For analyses of the mouse tumors, we used 35 *SB* tumor samples, comprising 13 histologically defined NEPC tumors and 22 histologically defined non-NEPC tumors (Table S1). The human tumor profiles were collected from two independent cohorts, namely, (1) the Beltran cohort (*n* = 49 patients), comprising 34 adenocarcinoma and 15 NEPC patients, as downloaded on November 12, 2020, from cBioPortal as FPKM measurements (Beltran et al., 2016) and (2) the SU2C cohort (*n* = 266 patients), comprising 210 non-NEPC, 22 NEPC patients and 34 unclassified tumors, as downloaded on October 4, 2019, from cBioPortal as FPKM measurements based on the 2019 SU2C-mCRPC dataset (Abida et al., 2019). For these analyses, we first generated an independent DGES for each mouse and patient-derived tumor sample by computing Z-scores obtained by dividing the difference between the expression of each gene in the sample and the mean expression in the associated cohort by the standard deviation.

Differentially active proteins were identified by analyzing each DGES using VIPER (Alvarez et al., 2016) for the human samples and metaVIPER (Ding et al., 2018) for the mouse samples, with the species-appropriate regulatory networks, see Vasciaveo et al. (2023). The mouse signatures were processed integrating both the human SU2C network and mouse prostate cancer network as described in Vasciaveo et al. (2023). Protein activity signatures for the mouse tumors are provided in Table S3 A and for the human tumors in Table S4 C and D.

To match the mouse *SB* prostate tumors to human prostate cancer patients, we used the OncoMatch algorithm (Vasciaveo et al., 2023). Specifically, we used the aREA algorithm (Alvarez et al., 2016) to assess enrichment of patient tumor-specific MRs—i.e., the top 50 most differentially active proteins in each human tumor, including the 25 most activated (25↑) and 25 most differentially inactivated proteins (25↓)—in proteins differentially active in the mouse *SB* tumors. For this purpose, mouse proteins were humanized (i.e., mapped to their human protein orthologs) before running aREA (Vasciaveo et al., 2023). Normalized enrichment scores (NES) were converted to P values and the Bonferroni method was used to correct for multiple hypothesis testing. The value of ${OM}_{Score}=-{\mathrm{Log}}_{10}\;\left(p-value\right)>5$ was used as a conservative statistical threshold to assess tumor/model fidelity. The OncoMatch data are provided in Table S4.

### Identification of CIS and CIS genes

Targeted genomic sequencing was performed to identify transposon insertion sites in *NPp53-SB(+)* prostate tumors following the protocol published in Janik and Starr (2013). We analyzed 74 tumors for which the *NPp53-SB(+) T2/Onc2* transposition was confirmed by PCR (Fig. S1 A). Genomic DNA was extracted and digested with restriction enzymes flanking the left (BfaI) or right (NlaIII) side of the transposon, as in Janik and Starr (2013). The resulting 148 samples were subjected to linker-mediated PCR (LM-PCR) as published in Janik and Starr (2013). Samples were prepared using the primers in Table S8, and the barcodes described in Table S5. Samples were sequenced using the Illumina HiSeq with 100 bp read (Azenta).

CIS, which are defined as regions of the genome having transposon insertions (i.e., integration sites) across multiple independent tumors at a significantly greater frequency than that expected by chance (Dupuy et al., 2005), were identified using the TAPDANCE software (version 3.1), which both maps insertion reads and computes statistically significant CIS (Sarver et al., 2012). Reads were mapped using the University California Santa Cruz indexed bowtie 1.2.3 compatible mm10 reference genome. All default parameters were used, and the $library percent, which discards insertion regions that are represented by a low number of reads, was set to 0.001 (i.e., insertion regions represented by lower than 0.001% of total reads assigned to the tumor are discarded). As noted above, since the *T2/Onc2* transposon is located on chromosome 1, insertions on this chromosome were disregarded in subsequent analysis to overcome complications related to local hopping (Liang et al., 2009).

This analysis identified 122 unique CIS (Table S6 A). For 121 of the 122 CIS, we identified 330 CIS-associated genes based on annotated genomic location within 20 kbp of the CIS. One CIS lacked any annotated gene feature within 20 kbp of the insertion. A list of the 330 CIS-associated genes together with the standardized TAPDANCE output is provided in Table S6 B. For each CIS-associated gene we also report the proportion of NEPC tumors calculated based on two-sided binomial test (stats) using p = proportion of neuroendocrine samples (27/74) for neuroendocrine versus adenocarcinoma.

The 330 CIS-associated genes were represented in a Circos plot, using the circlize (0.4.16) package. Strandedness was assessed from the raw TAPDANCE mapping output file, with the height of the dots representing each transposon’s read count. CIS were marked based on TAPDANCE output file (Table S6 A) and gene positions were acquired by bioMart (2.60.1) and plotted in the outer side of the Circos plot. Co-occurrence analysis of pairs of CIS-associated genes was performed using the somaticInteractions function using the maftools (2.20.0) package and is represented in the center of the Circos plot.

Lollipop plots for selected CIS-associated genes were generated using trackViewer (1.41.5) and GenomicRanges (1.57.1) packages. Gene structures were obtained from the mm10 assembly, using the TxDb.Mmusculus.UCSC.mm10.knownGene (3.10.0) package. Transposon insertion data from TAPDANCE output were plotted, with insertion heights representing read counts. In Figs. 5 and S3 and insertions in the same direction as gene transcription are shown with red arrows, opposite in green.

To identify genes where transposon insertions correlate with altered transcript structure, we performed two complementary analyses. First, genome-wide isoform switching analysis was conducted using IsoformSwitchAnalyzeR (version 1.16.0) (Vitting-Seerup and Sandelin, 2019). Salmon (version 1.9.0)-quantified transcript abundances were imported with GTF annotation (GENCODE version M25) and filtered for lowly expressed transcripts. Differential isoform usage between NEPC and non-NEPC conditions was tested using DEXSeq (version 1.56.0), with significance thresholds of q <0.05 and |ΔIF| >0.1. Protein domains were annotated using HMMER against the Pfam database. Results were intersected with CIS genes to identify candidates with both insertions and isoform switching. Second, fine-grained coverage analysis was performed for prioritized CIS genes with intronic insertions. RNA-seq reads were extracted from BAM files across gene bodies using Rsamtools (version 2.26.0), and coverage was compared between NEPC samples harboring insertions, SB-negative controls (*n* = 12), and SB-positive-non-NEPC controls (*n* = 10). Coverage was normalized within each group to enable comparison of relative patterns across the gene body.

To detect transposon-mRNA fusion transcripts, paired end RNA-seq reads were aligned to a composite reference genome consisting of mm10 (with the En2 locus masked) and the T2/Onc2 transposon sequence (2,163 bp) appended as chromosome “chrSB” as described in Temiz et al. (2016). Alignments were performed using HISAT2 (version 2.2.1) with splice-site annotations from GENCODE version M25. Transposon-genome fusion junctions were identified using the FUSION FINDER approach with default parameters. Detected fusions were cross-referenced with DNA-based CIS insertion coordinates to identify events where both DNA insertion and RNA fusion evidence were present. Data for all TAPDANCE analysis are provided in Table S6.

### Prioritization of CIS genes representing candidate NEPC regulators

Candidate MRs regulating genes differentially expressed in NEPC versus non-NEPC mouse *SB* tumors were identified as described above. CINDy was then used to prioritize candidate modulators of MR activity (Giorgi et al., 2014), based on the analysis of RNA-seq profiles from human primary prostate tumors from the TCGA patient cohort (*N* = 498; Cancer Genome Atlas Research, 2015) and metastatic CRPC biopsies from the SU2C patient cohort (*N* = 266; Abida et al., 2019). Briefly, for each *cMR* (as above), candidate modulator gene [*M*], and MR transcriptional target [*T*]—as inferred by the ARACNe algorithm (Basso et al., 2005)—we computed the statistical significance of the conditional mutual information I[cMR;T|M], using RNA-seq profiles from both human cohorts, independently. The statistical significance (P value) of each candidate modulator gene was assessed by integrating the P values across all of its targets, as described in Giorgi et al. (2014), and across cohorts. Statistical significance was assessed at P ≤ 0.05 (FDR corrected).

To prioritize CIS genes, we assessed each gene by integrating three independent statistics, including (1) P value as assessed by TAPDANCE analysis, (2) differential expression in NEPC versus non-NEPC samples, and (3) CINDy-predicted activity as NEPC MR modulators. The P values generated for each of these three analyses were integrated using Fisher’s method. We then ranked the list by their VIPER scores. The data are presented in Table 2 and Table S7.

### Analyses of SIRT1 expression in human prostate cancer

All studies involving human tissue specimens were performed according to protocols approved by the Human Research Protection Office and Institutional Review Board at the University of Bern (Project ID: 2019-00328 and 2022-00978). Only anonymized tissues were used, and patient consent was obtained. SIRT1 expression was evaluated in retrospectively collected tissue from patients with advanced prostate cancer (*N* = 7). Five patients harbored prostate cancer with neuroendocrine differentiation (5/7). From those, three patients (3/7) presented a small cell neuroendocrine carcinoma with known prostate cancer and two patients (2/7) harbored a prostate cancer with neuroendocrine differentiation. In the remaining two patients (2/7), an acinar adenocarcinoma of the prostate was found. SIRT1 (10E4) from #04-1557; Sigma-Aldrich was stained on the Leica BOND RX Automated Stainer. The dilution for SIRT1 was 1:200 in AB diluent from Biosystems, and it was incubated for 30 min at room temperature. The pretreatment was conducted with citrate epitope retrieval buffer for 30 min at 100°C. The detection was done with BOND Polymer Refine DAB detection kit #DS9800, including counterstain with hematoxylin. The percentage of tumor cells presenting a SIRT1 nuclear expression was reported.

### Functional analysis in human cell-based models

LNCaP cells (CRL-1740; ATCC) and 22Rv1 cells (CRL-2505; ATCC) were maintained in Roswell Park Memorial Institute medium (RPMI-1640; ATCC) with 10% fetal bovine serum (FBS) (Thermo Fisher Scientific) and HEK-293FT cells (R700-07; Invitrogen) were cultured in Dulbecco’s Modified Eagle Medium (DMEM) with 10% FBS (Thermo Fisher Scientific). Cells were authenticated by STR profiling and tested negative for Mycoplasma using the Universal Mycoplasma Detection Kit (#30-1012 K; ATCC).

Analyses of loss or gain of function in LNCaP and 22RV1 cells, respectively, was performed as described in Arriaga et al. (2024) and Papachristodoulou et al. (2021). For loss-of-function studies, we analyzed the consequences of silencing SIRT1 in LNCaP cells using two independent shRNAs based on LT3GEPIR T3G-GFP-(miR-E)-PGK-Puro-IRES-rtTA3 (#111177; Addgene) (Fellmann et al., 2013). The sequences of the shRNA are provided in Table S8. For gain-of-function studies, we analyzed the consequences of overexpression of SIRT1 in CRISPRa-engineered 22RV1 cells, as described (Arriaga et al., 2024). A non-targeting sgRNA (sgNT) and two individual sgRNAs targeting *SIRT1* (sgSIRT1 #1 and #2) were generated based on the protospacer sequences identified in the hCRISPRa-v2 library in Horlbeck et al. (2016), and subcloned into the sgRNA template pU6-sgRNA EF1Alpha-puro-T2A-BFP (#60955; Addgene). Primer sequences are provided in Table S8. Procedures for the generation and infection with lentivirus were done as in Arriaga et al. (2024) and Papachristodoulou et al. (2021).

For analyses in culture, cells were seeded at 2 × 10^5^ cells (22RV1) or 5 × 10^5^ cells (LNCaP) per 10 cm dish in RPMI + 10% FBS. The following day, cells were changed to RPMI phenol red-free + 5% charcoal-stripped serum (CSS) and grown in DMSO and ethanol or 1 mM dibutyryl cyclic-AMP (db-cAMP, STEMCELL Technologies) and 0.5 mM 3-isobutyl-1-methylxanthine (IBMX; Thermo Fisher Scientific).

Colony formation and proliferation assays were performed as described in Papachristodoulou et al. (2021). Briefly, for colony formation assays, 22RV1 cells (1 × 10^3^ cells) were seeded in triplicate in 6-well plates and grown for 12 days. Studies controlled for effects on cell proliferation following induction toward neuroendocrine differentiation. Colonies were visualized by crystal violet staining and quantified using ImageJ. To measure proliferation, we performed 3-(4,5-dimethylthiazol-2-yl)-2,5-diphenyltetrazolium bromide (MTT) proliferation assays. 1 × 10^4^ cells were seeded in triplicate in 96-well plates and grown up to 72 h. MTT-based proliferation was quantified by measuring OD at 560 nm in a Varioskan LUX multimode microplate reader (Thermo Fisher Scientific).

For analyses of tumor growth in vivo, 1 × 10^6^ 22RV1 cells in 30 μl of 50/50 PBS/Matrigel (Corning) were injected into the anterior prostate (AP) of immunodeficient Athymic Nude mice (Envigo) as described (Arriaga et al., 2024). Mice were euthanized at 40 days after injection, and tumors were collected and analyzed as described (Arriaga et al., 2024).

For all cell culture studies, validation of mRNA expression levels was performed by quantitative real-time PCR with QuantiTect SYBR Green PCR Kit (QIAGEN, Hilden, Germany), using *GAPDH* as an internal control. Relative expression levels were calculated using the 2^−^^ΔΔCT^ method, as described (Papachristodoulou et al., 2021). Sequences of the primers are provided in Table S8. Validation of protein levels was performed by western blot using total cell lysates extracted with radioimmunoprecipitation assay buffer, as described (Papachristodoulou et al., 2021). Antibodies described in Table S8.

### Functional analysis in mouse NEPC organoids

A mouse NEPC organoid line was generated from a YFP-lineage marked *NPp53* mouse having a rare NEPC tumor (Zou et al., 2017), using protocols developed to establish mouse prostate organoids (Chua et al., 2014; Giacobbe et al., 2025, *Preprint*). Briefly, YFP-marked cells were FACS sorted with the BV421/FITC channels. Sorted cells were resuspended in organoid culture media as described in Chua et al. (2014) (hepatocyte culture medium [Corning], 5% heat-inactivated charcoal-stripped fetal bovine serum [CS-FBS, Gibco], 1× GlutaMAX [Gibco, Thermo Fisher Scientific], 10 ng/ml epidermal growth factor [EGF, Corning], 10 µM Y-27632 ROCK inhibitor [STEMCELL Technologies], 100 nM dihydrotestosterone [DHT; Sigma-Aldrich], 5% Matrigel [Corning], and 1× antibiotic-antimycotic [Gibco]). Cells were seeded at a density of 5,000 cells per well onto ultralow-attachment 96-well plates (3474; Corning). The samples were monitored for organoid growth via fluorescence imaging for 7 days.

For passaging, mouse organoids were dissociated by digestion in 1 ml 0.25% Trypsin-EDTA (Gibco) for 10 min at 37°C; digestion was stopped by the addition of 2 ml modified Hank’s Balanced Salt Solution (HBSS; STEMCELL Technologies) supplemented with 2% FBS. The dissociated cells were collected by centrifugation at 350 *g* for 5 min at 4°C and resuspended in organoid culture medium, as above. Cells were counted using a Countess II Automated Cell Counter (Thermo Fisher Scientific) and plated at a seeding density of 5,000 cells/well onto ultralow-attachment 96-well plates (Corning). Organoid lines were established following five consecutive passages.

For histopathological characterization, organoids were harvested by centrifugation at 250 *g* × 5 min at 4°C, fixed in 1 ml 10% formalin at 4°C overnight (Thermo Fisher Scientific), and placed in 80 μl HistoGel (Epredia) before embedding. Paraffin-embedded organoids were sectioned (3 µm) and subjected to histopathological analysis and imaging as above. All antibodies and secondary antibodies are described in Table S8.

Analyses of loss-of-function in mouse organoids was performed as described in Giacobbe et al. (2025, *Preprint*). Briefly, we analyzed the consequences of silencing *Sirt1* in mouse organoids using two independent shRNAs based on LT3GEPIR T3G-GFP-(miR-E)-PGK-Puro-IRES-rtTA3 (#111177; Addgene) (Fellmann et al., 2013). The sequences of the shRNA are provided in Table S8. Upon infection of organoids with the LT3GEPIR T3G-GFP-(miR-E)-PGK-Puro-IRES-rtTA3 based lentiviruses, puromycin was added in the organoid culture medium (5 µg/ml) and cells were selected for 4 days. Following removal of the puromycin, doxycycline (0.5 µg/ml) was added for 2 consecutive days to induce shRNA-mediated silencing and was maintained in culture for 10 days. Tumor organoids were subjected to histopathological analysis and validation of mRNA expression levels was performed as above.

Functional analyses were performed using organoid lines that were infected with the lentiviruses as described above. For tumor growth assays, organoids (not dissociated) (equivalent to 2 × 10^5^ cells) were injected into the flank of male immunodeficient Athymic Nude mice (Envigo). To maintain shRNA expression, doxycycline was continuously provided in the drinking water at a concentration of 2 mg/ml and refreshed three times weekly. Tumors were monitored by caliper measurement twice weekly and tumor volumes were calculated using the formula [volume = (width)^2^ × length/2]. Mice were sacrificed when the tumor size reached 2,000 mm^3^ or if the body condition score of the host mice was <1.5 or if they exhibited signs of distress. At the time of sacrifice, YFP-positive allograft tumors were visualized by ex vivo fluorescence using an Olympus SZX16 microscope (Ex490–500/Em510–560 filter) and collected for histopathological analysis.

### Pharmacological inhibition of SIRT1

Studies were performed using a selective SIRT1 inhibitor, Selisistat EX-527 (purity: 99.78% from Selleckchem) resuspended in DMSO (Adams et al., 2024; Napper et al., 2005). To determine the dose of Selisistat that inhibits SIRT1 activity, we examined the overall acetylation of proteins using anti-acetyl Lysine antibodies (Abcam; ab22550) (Table S8). For studies in LNCaP cells, we seeded 5 × 10^5^ cells per 60 mm dish in RPMI + 10% FBS. The following day, cells were changed to RPMI phenol red-free + 5% CSS and treated with either DMSO and ethanol, or 1 mM db-cAMP (from 500 mM stock in 50/50 in ethanol/PBS) (STEMCELL Technologies), 0.5 mM IBMX (from 1 M stock in DMSO) (Thermo Fisher Scientific), or Selisistat EX-527 (from 50 mM stock in DMSO) (Selleckchem). For studies in NEPC organoids, we seeded cells from dissociated organoids at a density of 5,000 cells per well onto ultralow-attachment 96-well plates (3474; Corning). The following day, organoids were treated with either DMSO or Selisistat EX-527 (Selleckchem) for 10 days as above, and samples were subjected to validation of mRNA expression levels as described above.

### Statistical analysis

Statistical analysis was performed using Graphpad Prism software (version 9.4.1) and R Studio (version 1.3.1093), R (version 4.1.2). Survival curves for the *SB* mice were plotted based on Kaplan-Meier analysis and significance was calculated based on the log-rank test using the survival (3.5-5) and survminer (0.4.9) packages in R. Metastasis and NEPC frequencies were calculated and significance was evaluated based on the Fisher’s exact test in R. Tumor weight of *SB* tumors was analyzed and significance was calculated based on Welch’s two sample *t*-test in R. Analysis of Ki67 staining was performed using Graphpad Prism (version 9.4.1) and significance was calculated with one-way ANOVA with Tukey’s multiple comparisons test. Functional validation experiments were plotted using Graphpad Prism and significance was calculated based on one-way ANOVA with Dunnett’s multiple comparisons test for tumor weights, mRNA relative expression from cell lines, relative number of colonies from colony formation assay, and OD from MTT assay. Significance for the above-mentioned statistical tests was assumed for P < 0.05.

### Online supplemental material

Fig. S1 shows additional analyses of the prostate phenotype of *SB* mice (related to Fig. 2). Fig. S2 shows additional OncoMatch analyses (related to Fig. 4). Fig. S3 shows lollipop representation of selected CIS genes (related to Fig. 5). Fig. S4 shows additional analyses of selected CIS genes (related to Fig. 5). Fig. S5 shows additional validation studies in human prostate cancer cells (related to Figs. 6 and 8) (source files for Figs. S1 and 6). Table S1 shows summary of *SB* mouse phenotypes. Table S2 shows differential gene expression analysis in *SB* mice. (A) Differential gene expression of NEPC versus non-NEPC mouse *SB* tumors. (B) Relative expression levels of AR and Neuro markers from Labrecque et al. (2019). Table S3 shows protein activity signatures and cluster analyses of of mouse *SB* tumors. (A) Protein activity signatures from mouse *SB* tumors. (B) Protein activity of cluster analyses of mouse *SB* tumors. Table S4 shows oncomatch analysis between *SB* mice and prostate cancer patients. (A) OncoMatch analysis between *SB* mice and patients from the SU2C cohort. (B) OncoMatch analysis between *SB* mice and patients from the Beltran cohort. (C) SU2C protein activity. (D) Beltran 2016 protein activity. Table S5 shows *SB* barcodes. Table S6 shows a summary of CIS analyses and CIS-associated genes. (A) List of CIS determined by TAPDANCE analysis. (B) List of CIS-associated genes determined by TAPDANCE analysis. (C) List of all fusion events overlapping with a CIS region. (D) List of all genes with fusion events. (E) Isoform switching analyses of CIS-associated genes. (F) Domain details from isoform switching analyses of CIS-associated genes. (G) Full data from isoform switching analyses of CIS-associated genes. Table S7 shows prioritization of CIS-associated genes. Table S8 shows the key resources table.

## Supplementary Material

Table S1 shows summary of *SB* mouse phenotypes.

Table S2 shows differential gene expression analysis in *SB* mice. (A) Differential gene expression of NEPC versus non-NEPC mouse *SB* tumors. (B) Relative expression levels of AR and Neuro markers from Labrecque et al. (2019).

Table S3 shows protein activity signatures and cluster analyses of of mouse *SB* tumors. (A) Protein activity signatures from mouse *SB* tumors. (B) Protein activity of cluster analyses of mouse *SB* tumors.

Table S4 shows oncomatch analysis between *SB* mice and prostate cancer patients. (A) OncoMatch analysis between *SB* mice and patients from the SU2C cohort. (B) OncoMatch analysis between *SB* mice and patients from the Beltran cohort. (C) SU2C protein activity. (D) Beltran 2016 protein activity.

Table S5 shows *SB* barcodes.

Table S6 shows summary of CIS analyses and CIS-associated genes. (A) List of common insertion sites (CIS) determined by TAPDANCE analysis. (B) List of CIS-associated genes determined by TAPDANCE analysis. (C) List of all fusion events overlapping with a CIS region. (D) List of all genes with fusion events. (E) Isoform switching analyses of CIS-associated genes. (F) Domain details from isoform switching analyses of CIS-associated genes. (G) Full data from isoform switching analyses of CIS-associated genes.

Table S7 shows prioritization of CIS-associated genes.

Table S8 shows the key resources table.

SourceData F6 is the source file for Fig. 6.

SourceData FS1 is the source file for Fig. S1.

## Data Availability

RNA-seq expression profiling data have been deposited in the Gene Expression Omnibus (GEO) database (https://www.ncbi.nlm.nih.gov/geo/) with the following accession codes: GSE271066 (a description of all data from the study), GSE271054 (LM-PCR-based targeted genomic sequencing from NPp53-SB(+) mice), GSE271053 (mouse RNA-seq data from NP/NPp53-SB(−) and NP/NPp53-SB(+) mice). Code for all analyses is publicly available at https://github.com/alevax/sleeping-beauty-paper.
