# Supplementary material for: A forward genetic screen identifies Sirtuin1 as a driver of neuroendocrine prostate cancer
Source: J Exp Med. 2026 May 28;223(7):e20241484. doi: 10.1084/jem.20241484 (PMC13218332; doi:10.1084/jem.20241484)
Supplement: Table S8 — shows the key resources table. [file jem_20241484_tables8.docx]

**Table S8. Key Resources**

| REAGENT or RESOURCE | SOURCE | IDENTIFIER  (RRID) |
| --- | --- | --- |
| Antibodies | | |
| Rabbit monoclonal anti-AR (IHC/IF 1/200, WB 1/2,000) | Abcam | ab133273  (RRID:AB_11156085) |
| Mouse monoclonal anti-acetyl Lysine (IF 1/200) | Abcam | ab22550  (RRID:AB_447149) |
| Rabbit monoclonal anti-β-actin (WB 1/2,000) | Cell Signaling Technology | 4970  (RRID:AB_2223172) |
| Mouse monoclonal anti-INSM1 (IHC/IF 1/200) | Santa Cruz | sc-271408  (RRID:AB_10607955) |
| Rat monoclonal anti-Ki67 (IHC/IF 1/500) | eBiosciences | 14-5698-82  (RRID:AB_10854564) |
| Rabbit polyclonal anti-Pan-cytokeratin (IHC/IF 1/500) | Dako | Z0622  (RRID:AB_2650434) |
| Mouse monoclonal anti-SB-transposase (IHC/IF 1/200) | R&D Systems | MAB2798  (RRID:AB_573239) |
| Mouse monoclonal anti-Sirt1 (IHC/IF 1/100) | Sigma | 04-1557  (RRID:AB_1977495) |
| Rabbit polyclonal anti-Sirt1 (IHC/IF 1/200) | Sigma | 07-131  (RRID:AB_10067921) |
| Rat monoclonal anti-Sox2 (IHC/IF 1/50) | Invitrogen | 14-9811-82  (RRID:AB_11219471) |
| Mouse monoclonal anti-Synaptophysin (IHC/IF 1/500) | BD Transduction Laboratories | 611880  (RRID:AB_399360) |
| Rabbit monoclonal anti-Vimentin (IHC/IF 1/200) | Cell Signaling Technology | 5741  (RRID:AB_10695459) |
| Goat anti-Rabbit IgG (H+L) Cross-Adsorbed Secondary Antibody, Alexa Fluor™ 488 (IF 1/1,000) | Invitrogen | A11008  (RRID:AB_143165) |
| Goat anti-Mouse IgG (H+L) Highly Cross-Adsorbed Secondary Antibody, Alexa Fluor™ 488 (IF 1/1,000) | Invitrogen | A11029  (RRID:AB_2534088) |
| Goat anti-Rabbit IgG (H+L) Cross-Adsorbed Secondary Antibody, Alexa Fluor™ 555 (IF 1/1,000) | Invitrogen | A21428  (RRID:AB_2535849) |
| Goat anti-Rat IgG (H+L) Cross-Adsorbed Secondary Antibody, Alexa Fluor™ 555 (IF 1/1,000) | Invitrogen | A21434  (RRID:AB_2535855) |
| Amersham ECL Mouse IgG, HRP-linked whole Ab (from sheep) (WB 1/10,000) | Cytiva | NA931  (RRID:AB_772210) |
| Amersham ECL Rabbit IgG, HRP-linked whole Ab (from donkey) (WB 1/10,000) | Cytiva | NA934  (RRID:AB_772206) |
| Goat Anti-Rabbit IgG Antibody (H+L), Biotinylated (IHC 1/300) | Vector Labs | BA-1000  (RRID:AB_2313606) |
| Horse Anti-Mouse IgG Antibody (H+L), Biotinylated (IHC 1/300) | Vector Labs | BA-2000  (RRID:AB_2313581) |
| Goat Anti-Rat IgG Antibody (H+L), Biotinylated (IHC 1/300) | Vector Labs | BA-9400  (RRID:AB_2336202) |
| Bacterial and virus strains | | |
| NEB Stable Competent *E. coli* | New England Biolabs, Inc. | C3040H |
| Biological samples |  |  |
| Tissues from 7 prostate cancer patients | approved by the Ethical Committee Bern | Project ID: 2019-00328 and 2022-00978 |
| Chemicals, peptides, and recombinant proteins | | |
| Tamoxifen | Sigma | T5648 |
| cAMP | STEMCELL | 73884 |
| 3-Isobutyl-1-methylxanthine, IBMX | ThermoFisher Scientific | I5879-100MG |
| Selisistat (EX-527) (Purity: 99.78%) | Selleckchem | S1541 |
| Critical commercial assays | | |
| MagMAX-96 total RNA isolation kit | ThermoFisher Scientific | AM1830 |
| Deposited data | | |
| Description of the data in this study | GSE271066 |  |
| LM-PCR-based targeted genomic sequencing from NPp53-SB(+) mice | GSE271054 |  |
| Mouse RNASeq data from NP/NPp53-SB(—) and NP/NPp53-SB(+) mice | GSE271053 |  |
| Experimental models: Cell lines and organoids | | |
| Human: LNCaP cells | ATCC | CRL-1740  (RRID:CVCL_1379) |
| Human: 22Rv1 cells | ATCC | CRL-2505  (RRID:CVCL_1045) |
| Human: HEK-293FT cells | Invitrogen | R700-07  (RRID:CVCL_6911) |
| Mouse: primary tumor derived organoid line from the *NPp53* model | This study | N/A |
| Experimental models: Organisms/strains | | |
| Mouse: *Rosa26-LSL-SB11; T2/Onc2* (line *6113*) | NCI Mouse Repository, Frederick National Laboratory | 01XGA  (RRID:IMSR_NCIMR:01XGA) |
| Mouse: *NPp53 (Nkx3.1^CreERT2/+^; Pten^flox/flox^; Trp53^flox/flox^)* | Zou et al. (2017)  The Jackson Laboratory mouse repository | 033755  (RRID:IMSR_JAX:033755) |
| Mouse: Athymic Nude mice | Envigo | MGI:5652489  (RRID:MGI:5652489) |
| Oligonucleotides | | |
| ***Transposon excision PCR:*** |  |  |
| T2/Onc excision PCR forward:  TGTGCTGCAAGGCGATTA | Collier et al. (2005) |  |
| T2/Onc excision PCR reverse:  ACCATGATTACGCCAAGC | Collier et al. (2005) |  |
| ***LM-PCR - Linker sequences:*** |  |  |
| BfaI linker forward:  GTAATACGACTCACTATAGGGCTCCGCTTAAGGGAC | Janik and Starr (2013) |  |
| BfaI linker reverse:  Phos-TAGTCCCTTAAGCGGAG | Janik and Starr (2013) |  |
| NlaIII linker forward:  GTAATACGACTCACTATAGGGCTCCGCTTAAGGGACCATG | Janik and Starr (2013) |  |
| BfaI/NlaIII linker reverse:  Phos-GTCCCTTAAGCGGAGCC | Janik and Starr (2013) |  |
| ***LM-PCR - Primary PCR sequences:*** |  |  |
| Left side (BfaI) forward:  CTGGAATTTTCCAAGCTGTTTAAAGGCACAGTCAAC | Janik and Starr (2013) |  |
| Right side (NlaIII) forward:  GCTTGTGGAAGGCTACTCGAAATGTTTGACCC | Janik and Starr (2013) |  |
| Left/Right side reverse:  GTAATACGACTCACTATAGGGC | Janik and Starr (2013) |  |
| ***LM-PCR - Secondary PCR sequences:*** |  |  |
| Left side (BfaI) forward:  AATGATACGGCGACCACCGAGATCTACACTCTTTCCCTACACGACGCTCTTCCGATCTNNNNNNNNNNNNaAgtgtatgtaaacttccgacttcaa | Janik and Starr (2013) |  |
| Right side (NlaIII) forward:  AATGATACGGCGACCACCGAGATCTACACTCTTTCCCTACACGACGCTCTTCCGATCTNNNNNNNNNNNNaGgtgtatgtaaacttccgacttcaa | Janik and Starr (2013) |  |
| Left/Right side reverse:  CAAGCAGAAGACGGCATACGAGCTCTTCCGATCTAGGGCTCCGCTTAAGGGAC | Janik and Starr (2013) |  |
| **qPCR primers (mouse):** |  |  |
| Ar forward:  CGACTATTACTTTCCACCCCAG |  |  |
| Ar reverse:  TGCTGGCACATAGATACTTCTG |  |  |
| Gapdh forward:  AGGTCGGTGTGAACGGATTTG |  |  |
| Gapdh reverse:  TGTAGACCATGTAGTTGAGGTCA |  |  |
| ChgA forward:  CAGGCTACAAAGCGATCCAG |  |  |
| ChgA reverse:  GCCTCTGTCTTTCCATCTCC |  |  |
| Syp forward:  CATTCAGGCTGCACCAAGTG |  |  |
| Syp reverse:  TGGTAGTGCCCCCTTTAACG |  |  |
| Sirt1 forward:  GCCGCGGATAGGTCCATATA |  |  |
| Sirt1 reverse:  TCGAGGATCGGTGCCAAT |  |  |
| Ascl1 forward:  GCAACCGGGTCAAGTTGGT |  |  |
| Ascl1 reverse:  GTCGTTGGAGTAGTTGGGGG |  |  |
| Insm1 forward:  CTGGCGGCGTATCCGAATC |  |  |
| Insm1 reverse:  CCTGGCGACGGAACTTCTT |  |  |
| Sox2 forward:  TTTGTCCGAGACCGAGAAGC |  |  |
| Sox2 reverse:  CTCCGGGAAGCGTGTACTTA |  |  |
| Sox11 forward:  CGACGACCTCATGTTCGACC |  |  |
| Sox11 reverse:  GACAGGGATAGGTTCCCCG |  |  |
| Mycn forward:  ACCATGCCGGGGATGATCT |  |  |
| Mycn reverse:  ATCTCCGTAGCCCAATTCGAG |  |  |
| **qPCR primers (human):** |  |  |
| AR forward:  CTCCGCTGACCTTAAAGACATC |  |  |
| AR reverse:  TGCCCCCTAAGTAATTGTCCTT |  |  |
| CHGA forward:  CTGAACACAGGCAGCTTTCTA |  |  |
| CHGA reverse:  CAGTCAGGAGTTCTCAGCTTTC |  |  |
| GAPDH forward:  ACAACTTTGGTATCGTGGAAGG |  |  |
| GAPDH reverse:  GCCATCACGCCACAGTTTC |  |  |
| NEUROD1 forward:  GGTGCCTTGCTATTCTAAGACGC |  |  |
| NEUROD1 reverse:  GCAAAGCGTCTGAACGAAGGAG |  |  |
| SIRT1 forward:  TGGCAAAGGAGCAGATTAGTAGG |  |  |
| SIRT1 reverse:  CTGCCACAAGAACTAGAGGATAAGA |  |  |
| SYP forward:  TCAGTTCCGGGTGGTCAAG |  |  |
| SYP reverse:  AAGACCCATTGCAGCACCTT |  |  |
| MYCN forward:  CTGGGAACTGTGTTGGAG |  |  |
| MYCN reverse:  CGACTGAGGGCTTCTTTC |  |  |
| INSM1 forward:  CAGTGTGCGGAGAGTCGTT |  |  |
| INSM1 reverse:  ACCTGTCTGTTTTCGGATGG |  |  |
| ASCL1 forward:  CCCAAGCAAGTCAAGCGACA |  |  |
| ASCL1 reverse:  AAGCCGCTGAAGTTGAGCC |  |  |
| MYT1 forward:  ACTCCAGGCACCGAAGTTTAC |  |  |
| MYT1 reverse:  AGAGGCGTCCTTCACCTCA |  |  |
| TWIST1 forward:  CTTCTCGGTCTGGAGGATGG |  |  |
| TWIST1 reverse:  CTCCTTCTCTGGAAACAATGACA |  |  |
| SNAI1 forward:  TCGGAAGCCTAACTACAGCGA |  |  |
| SNAI1 reverse:  AGATGAGCATTGGCAGCGAG |  |  |
| RB1 forward:  CTCTCGTCAGGCTTGAGTTTG |  |  |
| RB1 reverse:  GACATCTCATCTAGGTCAACTGC |  |  |
| TP53 forward:  CAGCACATGACGGAGGTTGT |  |  |
| TP53 reverse:  TCATCCAAATACTCCACACGC |  |  |
| TWIST2 forward:  GCAAGATCCAGACGCTCAAGCT |  |  |
| TWIST2 reverse:  ACACGGAGAAGGCGTAGCTGAG |  |  |
| ONECUT2 forward:  GGAATCCAAAACCGTGGAGTAA |  |  |
| ONECUT2 reverse:  CTCTTTGCGTTTGCACGCTG |  |  |
| FOXA2 forward:  GGAGCAGCTACTATGCAGAGC |  |  |
| FOXA2 reverse:  CGTGTTCATGCCGTTCATCC |  |  |
| FOXM1 forward:  CGTCGGCCACTGATTCTCAAA |  |  |
| FOXM1 reverse:  GGCAGGGGATCTCTTAGGTTC |  |  |
| SOX11 forward:  AGCAAGAAATGCGGCAAGC |  |  |
| SOX11 reverse:  ATCCAGAAACACGCACTTGAC |  |  |
| **CRISPRa primers:** |  |  |
| Non-targeting forward:  ttgGGACCCTGACATGTATGTAGgtttaagagc | Horlbeck et al. (2016) |  |
| Non-targeting reverse:  ttagctcttaaacCTACATACATGTCAGGGTCCcaacaag | Horlbeck et al. (2016) |  |
| SIRT1 sgRNA #1 forward:  ttgGCCGAGTGCGCTTCCAGCCCgtttaagagc | Horlbeck et al. (2016) |  |
| SIRT1 sgRNA #1 reverse:  ttagctcttaaacGGGCTGGAAGCGCACTCGGCcaacaag | Horlbeck et al. (2016) |  |
| SIRT1 sgRNA #2 forward:  ttgGTCTACCGCTCCGCCTGGGCgtttaagagc | Horlbeck et al. (2016) |  |
| SIRT1 sgRNA #2 reverse:  ttagctcttaaacGCCCAGGCGGAGCGGTAGACcaacaag | Horlbeck et al. (2016) |  |
| **shRNA primers:** |  |  |
| SIRT1.1818 (SIRT1 sh1) – 97mer oligo:  tgctgttgacagtgagcgcTCAGCTGTTGGTCAAGACTAAtagtgaagccacagatgtaTTAGTCTTGACCAACAGCTGAttgcctactgcctcgga | Fellmann et al. (2013) |  |
| SIRT1.1849 (SIRT1 sh2) – 97mer oligo:  tgctgttgacagtgagcgcAAGGTTCATTTGTATGATAAAtagtgaagccacagatgtaTTTATCATACAAATGAACCTTttgcctactgcctcgga | Fellmann et al. (2013) |  |
| Sirt1.1607 (Sirt1 sh1) – 97mer oligo:  tgctgttgacagtgagcgcAACAACAATGTTAATGATTTAtagtgaagccacagatgtaTAAATCATTAACATTGTTGTTTTGCCTACTGCCTCGGA | Fellmann et al. (2013) |  |
| Sirt1.2826 (Sirt1 sh2) – 97mer oligo:  tgctgttgacagtgagcgcTCAGTTGTTCTAGAAACAATAtagtgaagccacagatgtaTATTGTTTCTAGAACAACTGAATGCCTACTGCCTCGGA | Fellmann et al. (2013) |  |
| Recombinant DNA | | |
| pHRdSV40-dCas9-10xGCN4_v4-P2A-BFP | Addgene | 60903 (RRID:Addgene_60903) |
| pHRdSV40-scFv-GCN4-sfGFP-VP64-GB1-NLS | Addgene | 60904 (RRID:Addgene_60904) |
| pU6-sgRNA EF1Alpha-puro-T2A-BFP | Addgene | 60955 (RRID:Addgene_60955) |
| LT3GEPIR T3G-GFP-(miR-E)-PGK-Puro-IRES-rtTA3 | Addgene | 111177 (RRID:Addgene_111177) |
| psPAX2 | Addgene | 12260 (RRID:Addgene_12260) |
| pMD2.G | Addgene | 12259 (RRID:Addgene_12259) |
| Software and algorithms | | |
| Graphpad Prism v10.2.2 for MacOS | GraphPad Software, Boston, Massachusetts USA | [www.graphpad.com](http://www.graphpad.com)  (RRID:SCR_002798) |
| Fiji Is Just ImageJ (Fiji) v2.9.0 | Schindelin et al. (2012) | <https://imagej.net/software/fiji/>  (RRID:SCR_002285) |
| R | <https://www.r-project.org/> | R version 4.3.1 (2023-06-16)  (RRID:SCR_001905) |
| R Studio | <https://posit.co/> | 2023.12.1 Build 402  (RRID:SCR_000432) |
| TAPDANCE v3.1 | <https://sourceforge.net/p/tapdancebio/home/Home/> | https://github.com/mat10d/TAPDANCEV2 |
| DESeq2 | <https://www.bioconductor.org/packages/release/bioc/html/DESeq2.html> | v1.41.2 (RRID:SCR_015687) |
| VIPER | <https://www.bioconductor.org/packages/release/bioc/html/viper.html> | v1.36.0 |
| ARACNe-AP | <https://github.com/califano-lab/ARACNe-AP> | Master branch (RRID:SCR_002180) |
| CINDy | <https://califano.c2b2.columbia.edu/mindy2-cindy> | standalone |
| edgeR | <https://bioconductor.org/packages/release/bioc/html/edgeR.html> | v4.0.16 (RRID:SCR_012802) |
| ComplexHeatmap | <https://bioconductor.org/packages/release/bioc/html/ComplexHeatmap.html> | v2.18.0 (RRID:SCR_017270) |
| Original Code | <https://github.com/alevax/sleeping-beauty-paper> | Master branch |
| Other | | |
|  |  |  |

**References cited:**

Collier, L.S., C.M. Carlson, S. Ravimohan, A.J. Dupuy, and D.A. Largaespada. 2005. Cancer gene discovery in solid tumours using transposon-based somatic mutagenesis in the mouse. *Nature* 436:272–276. https://doi.org/10.1038/nature03681

Fellmann, C., T. Hoffmann, V. Sridhar, B. Hopfgartner, M. Muhar, M. Roth, D.Y. Lai, I.A.M. Barbosa, J.S. Kwon, Y. Guan, et al. 2013. An optimized microRNA backbone for effective single-copy RNAi. *Cell Rep.* 5:1704–1713. https://doi.org/10.1016/j.celrep.2013.11.020

Horlbeck, M.A., L.A. Gilbert, J.E. Villalta, B. Adamson, R.A. Pak, Y. Chen, A.P. Fields, C.Y. Park, J.E. Corn, M. Kampmann, and J.S. Weissman. 2016. Compact and highly active next-generation libraries for CRISPR-mediated gene repression and activation. *Elife* 5:e19760. https://doi.org/10.7554/eLife.19760

Janik, C.L., and T.K. Starr. 2013. Identification of Sleeping Beauty transposon insertions in solid tumors using linker-mediated PCR. *J. Vis. Exp.* 50156. https://doi.org/10.3791/50156

Schindelin, J., I. Arganda-Carreras, E. Frise, V. Kaynig, M. Longair, T. Pietzsch, S. Preibisch, C. Rueden, S. Saalfeld, B. Schmid, et al. 2012. Fiji: an open-source platform for biological-image analysis. *Nat. Methods* 9:676–82. https://doi.org/10.1038/nmeth.2019

Zou, M., R. Toivanen, A. Mitrofanova, N. Floch, S. Hayati, Y. Sun, C. Le Magnen, D. Chester, E.A. Mostaghel, A. Califano, M.A. Rubin, M.M. Shen, and C. Abate-Shen. 2017. Transdifferentiation as a Mechanism of Treatment Resistance in a Mouse Model of Castration-Resistant Prostate Cancer. *Cancer Discov.* 7:736–749. https://doi.org/10.1158/2159-8290.CD-16-1174
