# Supplementary figures and images for: A forward genetic screen identifies Sirtuin1 as a driver of neuroendocrine prostate cancer
Source: J Exp Med. 2026 May 28;223(7):e20241484. doi: 10.1084/jem.20241484 (PMC13218332; doi:10.1084/jem.20241484)

# Source data for Figure 6F

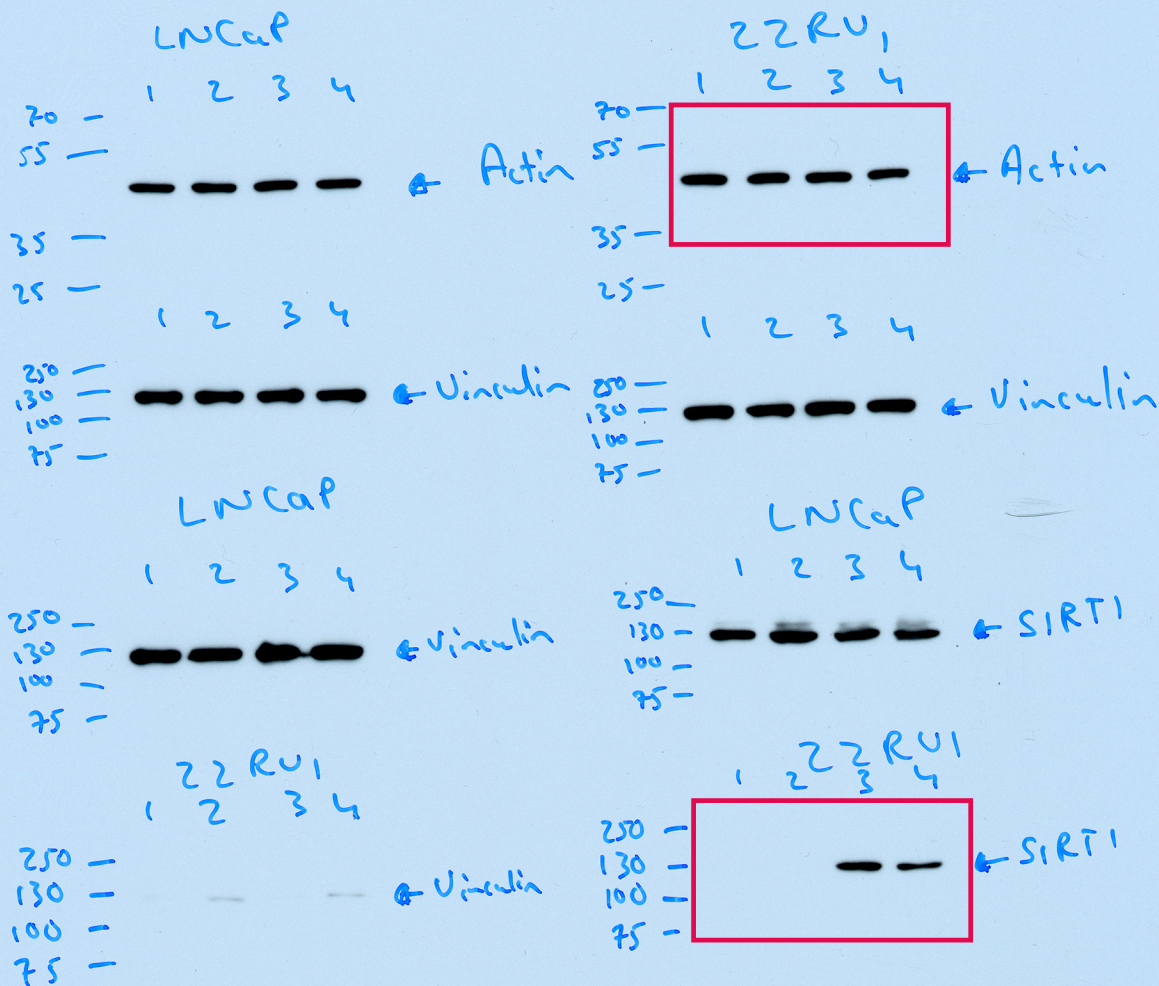

|   | LNCaP     | 22RV1 |
|---|-----------|-------|
| 1 | Day 0 Ctl | Ctl   |
| 2 | Day 7 Ctl | Ctl   |
| 3 | 7 Sg1     | Sg1   |
| 4 | 7 Sg2     | Sg2   |

Supplement: SourceData F6 — is the source file for Fig. 6. [file jem_20241484_sourcedataf6.pdf]

# Source Data for Figure S1A

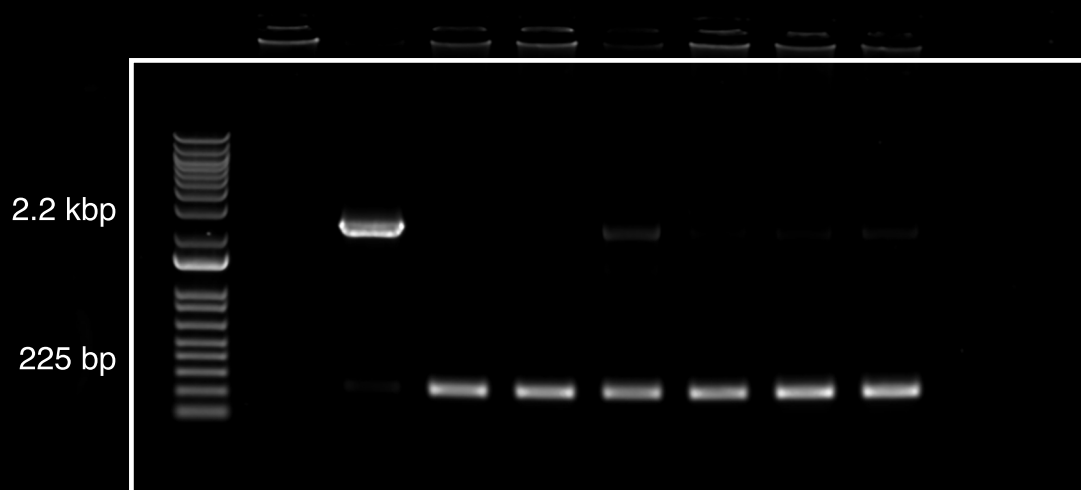

Supplement: SourceData FS1 — is the source file for Fig. S1. [file jem_20241484_sourcedatafs1.pdf]
